# Diffusion-Limited Kinetics of Isovalent Cation Exchange in III–V Nanocrystals Dispersed in Molten Salt Reaction Media

**DOI:** 10.1021/acs.nanolett.2c01699

**Published:** 2022-08-11

**Authors:** Aritrajit Gupta, Justin C. Ondry, Min Chen, Margaret H. Hudson, Igor Coropceanu, Nivedina A. Sarma, Dmitri V. Talapin

**Affiliations:** † Department of Chemistry, James Franck Institute, and Pritzker School of Molecular Engineering, University of Chicago, Chicago, Illinois 60637, United States; ‡ Center for Nanoscale Materials, Argonne National Laboratory, Argonne, Illinois 60439, United States

**Keywords:** alloyed III−V nanocrystals, cation exchange in molten salts, diffusion kinetics, diffraction simulations, HRTEM image analysis

## Abstract

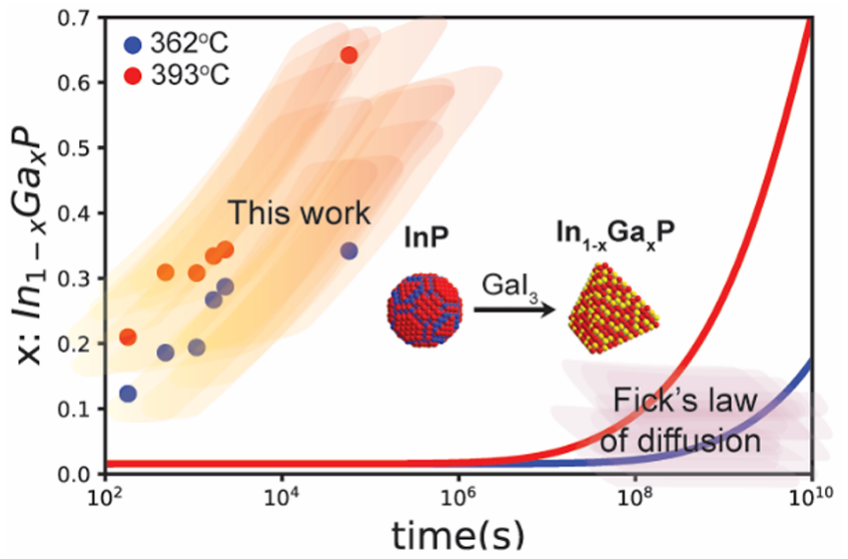

The goal of this work is to determine the kinetic factors that govern isovalent cation exchange in III–V colloidal quantum dots using molten salts as the solvent and cation source. We focus on the reactions of InP + GaI_3_→ In_1–*x*_Ga_*x*_P and InAs + GaI_3_→ In_1–*x*_Ga_*x*_As to create technologically important ternary III–V phases. We find that the molten salt reaction medium causes the transformation of nearly spherical InP nanocrystals to tetrahedron-shaped In_1–*x*_Ga_*x*_P nanocrystals. Furthermore, we determine that the activation energy for the cation exchange reaction is 0.9 eV for incorporation of Ga into InP and 1.2 eV for incorporation of Ga into InAs, both much lower than the measured values in bulk semiconductors. Next, we use powder XRD simulations to constrain our understanding of the structure of the In_1–*x*_Ga_*x*_P nanocrystals. Together our results reveal several important features of molten salt-mediated cation exchange and provide guidance for future development of these materials.

Colloidal quantum dots (QDs) constitute an important class of optoelectronic materials widely explored for display applications^1−3^ To date, colloidal quantum dots made of II–VI materials have shown the best optical performance in terms of near-unity photoluminescence quantum yield and narrow emission line width.^4−6^ The same level of performance has not been universally achieved for III–V colloidal nanocrystals. There are several reasons, however, to develop colloidal routes to III–V nanocrystals including lower toxicity^7^ and impressive optoelectronic performance achieved for nanostructures grown by CVD and MBE methods.^8−11^ Synthetic efforts to improve solution-synthesized III–V materials face several challenges. For example, typical precursors are very reactive,^12,13^ making it difficult to control nucleation and growth.^13−16^ III–V chemical bonds are predominantly covalent,^12,17,18^ making high-temperature processing necessary. In addition, Ga and Al are extremely oxophilic,^19,20^ making colloidal preparation of their pnictide phases difficult. Thus far, only the In–V (V = P, As, Sb) phases have been synthesized via traditional colloidal routes with reasonable material quality.^21−27^

In recent works, our group has addressed key challenges related to III–V semiconductors by introducing high-temperature molten salt annealing (>400 °C) and molten salt-mediated cation exchange to prepare ternary III–V phases. Initially, we used InP(As) nanocrystals capped with sulfide ligands and processed them in a mixture of LiBr/KBr/CsBr + GaI_3_ forming In_1–*x*_Ga_*x*_P(As).^28^ We recently explored the role of nanocrystal surface chemistry and gallium halide additives on the reactivity of InP nanocrystals in LiBr/KBr/CsBr and LiI/KI eutectic mixtures. These observations indicate that in the absence of added GaI_3_, chalcogenide surface ligands ((NH_4_)_2_S, Li_2_Se, (DDA)_2_S, etc.) were key to suppressing undesirable ripening at elevated temperatures.^29^ In addition, we found that nanocrystals capped with Lewis acid ligands^30^ (GaCl_3_, InCl_3_, etc.) were stable against undesirable ripening or decomposition only in the presence of excess GaI_3_. This procedural modification avoids concerns regarding the presence of chalcogenide atoms, which may deleteriously affect their optical performance.^31^

In this work, we build upon our success using Z-type inorganic gallium halide ligands for molten salt-mediated cation exchange.^29^ We replace the Lewis basic alkali halide salts with Lewis acidic salts consisting of GaI_3_ and KI mixtures, resulting in qualitatively better colloidal stability of the nanocrystals. Next, we use this chalcogenide-free molten salt system to systematically study the effect of cation exchange on particle morphology and the kinetics of the In-to-Ga replacement.^32^ We find that the activation energy of the rate-determining step in the cation exchange process is considerably lower (∼1 eV) compared to the activation energy measured for self-diffusion in the corresponding bulk systems.^33−35^ Finally, we use powder X-ray diffraction simulations of model nanocrystal structures to further constrain our understanding of the structure, composition, and heterogeneity of the In_1–*x*_Ga_*x*_P nanocrystal systems and show that X-ray diffraction methods have significant limitations in their ability to discern important classes of defects. We thus propose an approach for the analysis of compositional variation using high-resolution transmission electron microscopy as an alternative tool of structural interrogation.

We synthesized InP QDs using two distinct synthetic methods to prepare sphere- and tetrahedron-shaped InP. Sphere-shaped InP QDs were synthesized from InCl_3_ and (TMS)_3_P in trioctylphosphine (TOP) and trioctylphosphine oxide (TOPO) by an adaptation of the method developed by Mićić et al.^36,37^ Tetrahedron-shaped InP QDs were synthesized from InCl_3_ and (NMe_2_)_3_P in oleylamine following the methods developed by Hens et al.^38,39^ After size selective precipitation, we obtained sphere-shaped particles (Figure S1A,B) and tetrahedron-shaped particles with relatively narrow size distributions (Figure S1C,D). The distinct morphologies of the sphere- and tetrahedron-shaped nanocrystals provide an opportunity to explore the effect the molten salt annealing has on the shape and surface termination of the particles, as the reaction scheme in Figure 1A suggests. The photographs in Figure 1B show well-dispersed particles in the solidified molten salt matrix, consistent with our previously determined principles for colloidal stability.^40,41^ TEM images of the sphere-shaped InP QDs show little evidence of particle faceting (Figure 1C), whereas amine/chloride passivated InP QDs show faceted particles with triangular projections consistent with a tetrahedron shape (Figure 1F). Following cation exchange, the In_1–*x*_Ga_*x*_P QDs produced from either sphere- or tetrahedron-shaped nanocrystals both show faceted, triangular shapes in TEM images (Figures 1D and 1G, respectively). This suggests that the QD surface can recrystallize under the molten salt cation exchange conditions at ∼400 °C. The preference for tetrahedral morphology in the molten iodide matrix is supported by calculations that indicate favorable interactions between halide ions and the InP (111) surface facets, suggesting tetrahedral particles are the thermodynamically favored shapes of III–V nanocrystals in molten halide salts.^42^ Evidently, the reaction zone of the pertinent molten salt cation exchange process extends beyond the dimension of these InP nanocrystals.^43−45^ The change in morphology for initially spherical particles also suggests the phosphide sublattice is mobile, which may have important consequences related to the kinetics of the In-to-Ga cation exchange discussed below. This demonstrates that the process is not a simple replacement of In by Ga within a rigid phosphide lattice. Instead, the phosphide lattice reconfigures during the reaction as well, setting it apart from the low-temperature exchange reactions of ionic nanocrystals in which cations replace each other in a rigid anion lattice. Cation exchange in both sphere- and tetrahedron-shaped InP QD samples yields In_1–*x*_Ga_*x*_P QDs, as evidenced by a shift of the XRD peaks to higher angles, indicating a decreased lattice constant (Figure 1E,H) and a corresponding blue-shift in the absorbance onset (Figure S2). We note that the sphere-to-tetrahedron shape change is not expected to drastically alter XRD peak intensity ratios (Figure S13). Based on the lattice constants measured with XRD, we estimate the QD composition to be In_0.66_Ga_0.34_P for the initially spherical particles and In_0.58_Ga_0.42_P for the initially tetrahedron-shaped particles.

**Figure 1:**
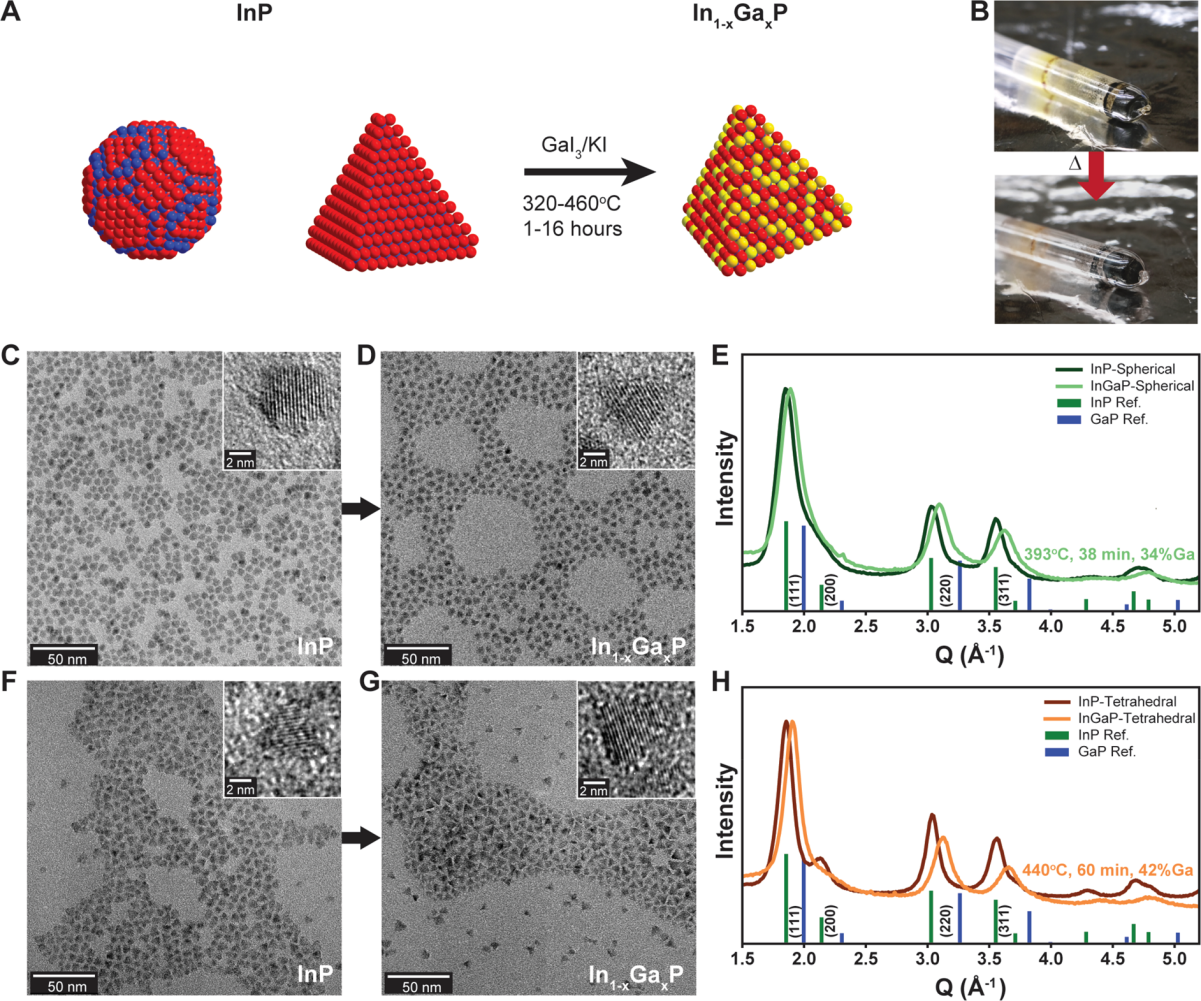
(A) Reaction scheme outlining the transformation of sphere- and tetrahedron-shaped InP nanocrystals into tetrahedron-shaped In_1–*x*_Ga_*x*_P nanocrystals. (B) Representative photographs of InP nanocrystals dispersed in GaI_3_/KI [65:35 mol %] eutectic molten salt (top) and the resulting In_1–*x*_Ga_*x*_P after annealing (bottom). (C–E) TEM images of sphere-shaped InP nanocrystals before (C) and after (D) annealing in the molten salt reaction medium with corresponding powder X-ray diffraction patterns (E). (F–H) TEM images of tetrahedron-shaped InP nanocrystals before (F) and after (G) annealing in the molten salt reaction medium with corresponding powder X-ray diffraction patterns (H).

The chemical bonding in III–V semiconductors is predominantly covalent.^12,17,18^ As a result, cation diffusion in bulk InP or GaP crystals is slow, characterized by a large activation energy and small diffusion coefficients. Studies on the diffusion of Ga^3+^ into bulk InP at the temperature ranges considered here are unavailable, so instead we extrapolate the reported self-diffusion coefficients to our working temperatures.^33,34^ Based on this, the self-diffusion coefficient would be on the order of 10^–24^ cm^2^/s for InP at our working temperature,^29^ requiring ∼32 years to incorporate 50% Ga into a 6.25 nm particle at 400 °C. This would indicate that Fickian diffusion for In-to-Ga cation exchange would be extremely slow at present reaction conditions. Yet we observe substantial alloying even at low temperatures, indicating that the pertinent mode of mass transport during molten salt mediated cation exchange in III–V nanocrystals has much lower barriers to Ga incorporation than in bulk systems. Previously, we hypothesized that three distinct steps exist in isovalent cation exchange processes in InP nanocrystals,^29^ the final step being the introduction of Ga^3+^ cations into the lattice with simultaneous expulsion of In^3+^ cations. This step is likely the slowest and rate-determining step. Modeling this final phase within the analytical framework of the solution to Fick’s second law of diffusion for spherical particles allows us to gain crucial insight into the mechanistic pathways of In-to-Ga cation exchange occurring under diffusion-limited control.

We start with spherical InP nanocrystals with ∼6.25 nm average diameter. After GaI_3_ ligand exchange, the particles were dispersed in a eutectic GaI_3_/KI molten salt matrix at 240 °C for 1 h, sealed inside a quartz ampule under vacuum, and annealed at different combinations of time and temperature by using a custom-built shaking furnace (Figure S3). After cation exchange, alloyed In_1–*x*_Ga_*x*_P nanocrystals were recovered with oleic acid and oleylamine ligands (OA/OAm) in toluene. The resulting colloidal solutions of nanocrystals in nonpolar solvents were characterized by using PXRD (Figure 2A,B). As expected, we observe the shift of diffraction peaks to higher momentum transfers (*Q* = 4π sin(θ)/λ, where 2θ is the Bragg angle and λ is the X-ray wavelength for the conventional 2θ axis; see Figure S4) for longer reaction times and higher reaction temperatures, consistent with the decrease in lattice parameter expected for higher gallium incorporation. We performed Le Bail refinement on each diffraction pattern^46^ to estimate lattice constants and calculated the gallium composition by linear interpolation from the pure bulk phases. An additional sample was prepared without the high-temperature annealing step, allowing us to estimate the contribution of the intermediate surface exchange step toward alloying at about ∼5%. We found that annealing spherical InP nanocrystals at 393 °C resulted in nanocrystals with gallium content ranging from 25% to 60%, with increasing time resulting in increased gallium incorporation (Figure 2A,B). This trend occurs simultaneously with a continuous blue-shift of the excitonic absorption feature with increasing gallium content (Figure S5). TEM images of these samples show increasingly distinct faceting with longer annealing durations (Figure S6). In addition, the faceting develops even within the shortest (3 min) annealing time, indicating that the disruption and subsequent recrystallization of the phosphide sublattice happens rather rapidly at 393 °C. Notably, the average Scherrer sizes of these nanocrystals are smaller than the corresponding SAXS and TEM estimates (Figure S1A,B). The tetrahedron-shaped In_1–*x*_Ga_*x*_P nanocrystals prepared with 1 h of annealing time have an average edge length of ∼8.5 nm via TEM (Figure S6), whereas the Scherrer estimate of the effective radius is 3.4–3.6 nm. We attribute this discrepancy to the presence of stacking faults and crystal twinning, as discussed in a later section. An annealing temperature of 362 °C yields similar trends (Figure S7) where an initially quick increase in gallium content is followed by slower incorporation at long times.

**Figure 2:**
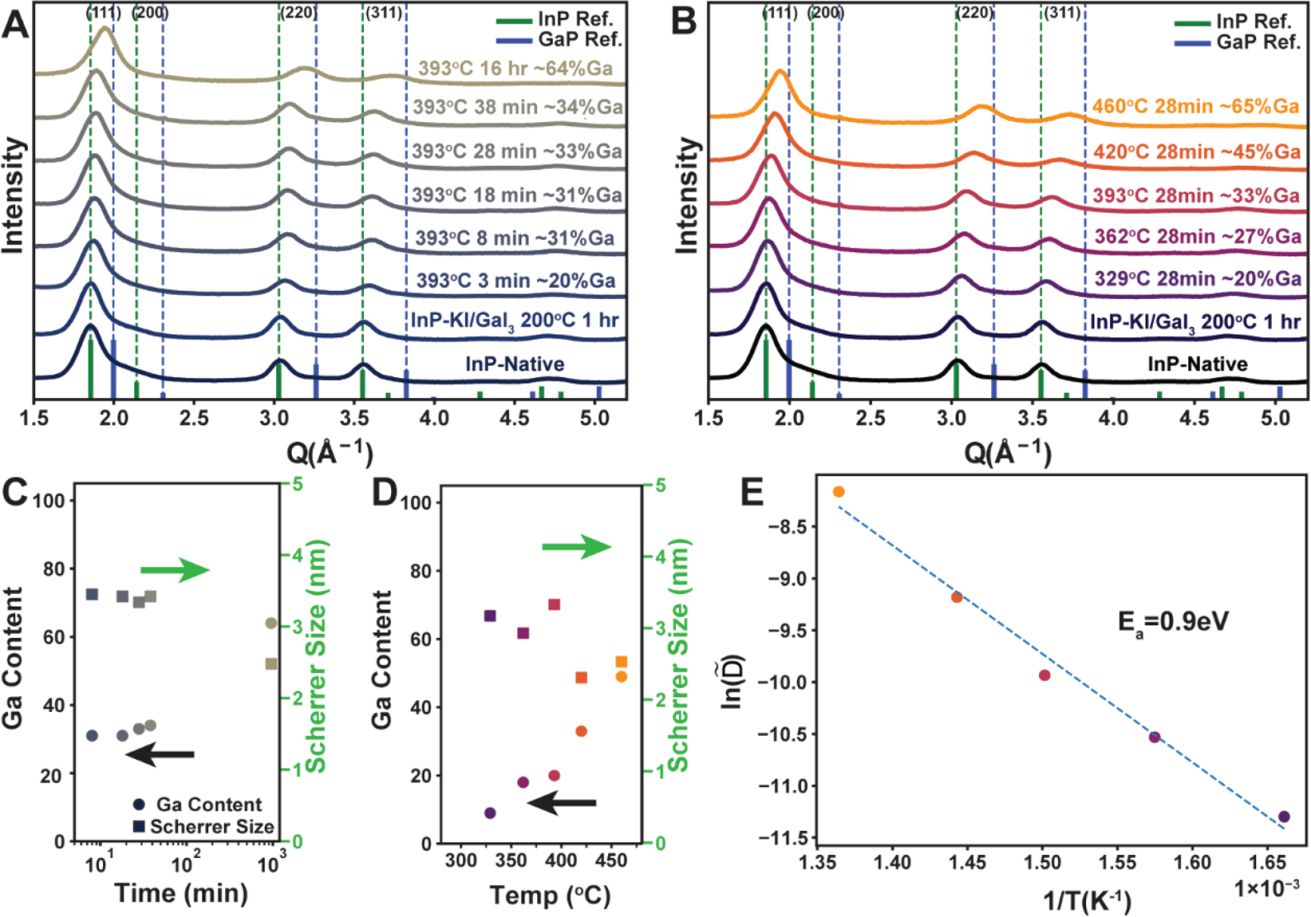
Powder XRD patterns for ∼6.25 nm InP nanocrystals annealed in GaI_3_/KI [65:35 mol %] for different times (A) and temperatures (B). Extracted gallium content (circles) and Scherrer size (squares) for the time (C) and temperature (D) series. Arrhenius plot of the apparent diffusion coefficient measured as a function of temperature with the extracted activation energy (E).

Next, we explored the temperature dependence of gallium incorporation by annealing the samples for a fixed duration at different temperatures (Figure 2B). From this, we extract an effective interdiffusion coefficient *D̃* at each of these annealing temperatures for a reaction duration of *t* and extent of conversion *x* by utilizing the analytical solution to Fick’s second law of diffusion for a spherical particle of radius *R* (see Annexure, Supporting Information).^47^
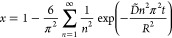
The gallium content increases monotonically with longer durations of annealing and increasing temperatures according to Figure 2C,D. As hypothesized previously, all the apparent interdiffusion coefficients extracted from the temperature series data are many orders of magnitude larger than their self-diffusion counterpart extrapolated from the available bulk data. Assuming an Arrhenius dependence of the apparent diffusion coefficients on temperature, we were able to estimate the activation barrier toward cation exchange, *E*_a_ ∼ 0.9 eV (∼87 kJ/mol), as noted in Figure 2E. These numbers are significantly lower than the activation barrier for self-diffusion previously reported in bulk III–V semiconductors, e.g., 3.85 eV for In in bulk InP,^33,34^ but similar in magnitude to prior reports across different II–VI and IV–VI nanocrystalline systems.^45,48,49^ Several high-diffusivity pathways could be invoked to explain this deviation. As noted previously, reorganization of the phosphide sublattice into a thermodynamically favored morphology is expected to generate defects and thus accelerate gallium diffusion. Additionally, these nanocrystals have stacking faults and twin defects (discussed later) which are known to be important low-temperature diffusion pathways.^50^

To test the generality of these observations, we repeated annealing experiments on smaller InP nanocrystals with an average diameter of 4 nm (Figure S8). The particles were processed similarly. The time and temperature series data under different annealing conditions are given in Figure S9A–C. We observe qualitatively similar trends, in that longer reaction times and higher temperatures result in higher gallium concentration. The resulting Arrhenius plot deviates significantly from linearity, suggesting competing pathways at different temperatures (Figure S9D). These smaller InP particles have a ∼60% higher surface-to-volume ratio relative to the ∼6.25 nm particles described before. Consequently, the surface exchange or surface recrystallization processes are expected to contribute more to the kinetics in these systems.^48^

Next, we explore the kinetics of cation exchange in InAs nanocrystals upon annealing in a KI/GaI_3_-based molten salt. Figure 3A and B shows the resulting powder X-ray diffraction patterns for InAs nanocrystals after high-temperature annealing under different conditions. Increased time and temperature led to greater shifts of the XRD peak positions to higher *Q* values, consistent with increased gallium incorporation. We quantified the particles’ composition and size by fitting the peaks to pseudo-Voigt functions and used extracted peak position and width to calculate the composition and Scherrer crystallite size, respectively (Figure 3C,D). Again, we find a monotonic increase in gallium content with both time and temperature. With regards to domain size, we see a slight increase in size as samples are annealed for a longer time (Figure 3C). Notably, treating InAs nanocrystals for 16 h at 400 °C results in the incorporation of over 80% Ga, suggesting cation exchange may be a viable route to nearly pure GaAs, a material which has been difficult to prepare by direct colloidal synthesis.^17^

**Figure 3:**
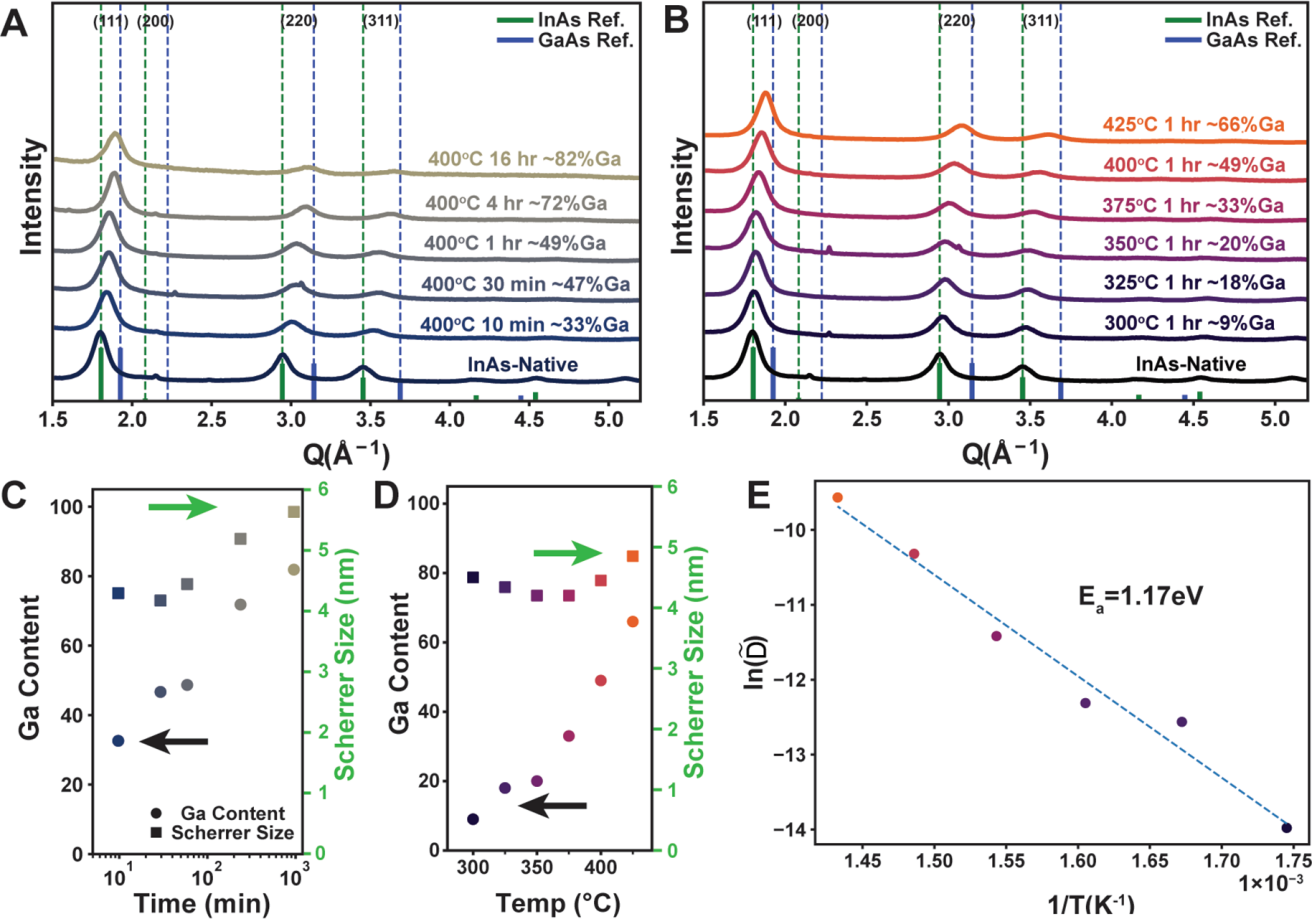
Powder XRD patterns for ∼4 nm InAs nanocrystals annealed in 1:1 KI/GaI_3_ for different times (A) and temperatures (B). Extracted gallium content (circles) and Scherrer size (squares) for the time (C) and temperature (D) series. Arrhenius plot of the apparent diffusion coefficients measured as a function of temperature with the extracted activation energy (E).

We measured the apparent diffusion coefficients as a function of temperature (Figure 3E), which followed an Arrhenius relationship with an associated activation energy of 1.2 eV. The larger activation energy leads to a stronger temperature dependence for gallium incorporation in InAs compared to InP. Again, the measured activation energy is much lower than the corresponding value measured for self-diffusion of Ga in either bulk GaAs (4.24 eV^35^ to 5.60 eV^33^) or bulk GaP (4.5 eV).^35^ These results indicate that significantly lowered activation energies for cation exchange in III–V nanocrystals using molten salts may be quite general.

Thus far, we have used the shift of the PXRD peak positions to determine the average lattice parameter of the nanocrystal sample and a subsequent linear interpolation of the lattice parameter/composition relationship (Vegard’s law) to determine the gallium content. For colloidal nanocrystals, other factors can also affect the observed lattice parameter, such as strain resulting from the radial variation of particle composition, as observed in a core/shell morphology.^51−53^ To this end, we aim to elucidate additional information from powder XRD to better constrain our understanding of the structure of these materials. We begin by simulating the powder XRD pattern (using the Debye formula) for a series of ideal zinc blende In_1–*x*_Ga_*x*_P nanocrystals (Figure 4A). Expectedly, we see shifts of the XRD peak positions to larger *Q* values according to the variation of the composition. In addition, we observe a slight decrease in the ratio of the (200) and (111) peak intensities (i.e., *I*_(200)_/*I*_(111)_ decreases) (see Figure S11 for quantification). This is expected based on the structure factor (*F*) for the (200) peak of a zinc blende material: *F*_(200)_ = 4(*f*_P_ – *f*_Ga/In_). Because the peak intensity, proportional to |*F*|^2^, is determined by the difference of the atomic scattering factors (*f*) of the anion and cation, the relative intensity of the (002) peak may provide an independent check of the gallium content. For In_1–*x*_Ga_*x*_As nanocrystals, the scattering factor of As is closer in magnitude to that of In and Ga, so the ratio of the (200) and (111) peak intensities changes to a smaller extent when *x* varies from 0 to 1 (Figure S12). For this reason, we focus on analyzing the In_1–*x*_Ga_*x*_P system for the remaining discussion.

**Figure 4:**
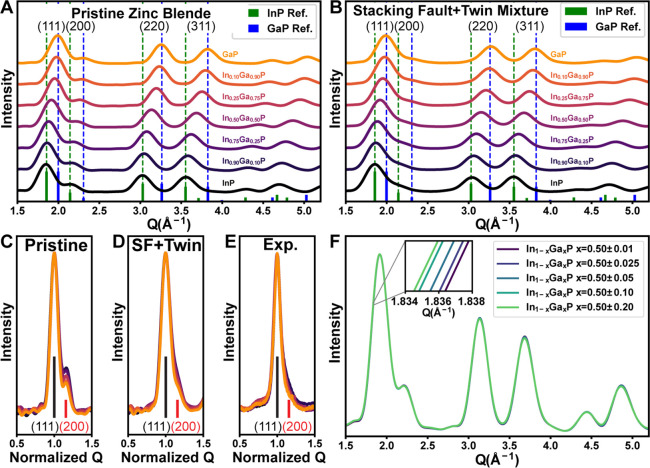
Simulated powder XRD patterns for (A) pristine zinc blende In_1–*x*_Ga_*x*_P nanocrystals and (B) In_1–*x*_Ga_*x*_P nanocrystals with stacking disorder (mixture of twins and stacking faults). Comparison of the (111) and (200) peak intensities without the peak shifts due to lattice parameter change, by normalizing the scattering vector to 1 for the (111) peak position: for the simulated pristine InP (C), including stacking fault + twin (D) and the experimental diffraction (E). The color scheme used in (C) and (D) corresponds to the same color scheme used in (A) and (B), respectively. For the experimental data in (E), the color scheme used is the same as in Figure 2B. The reference stick pattern is for pristine InP. (F) Simulated powder XRD patterns for an ensemble of In_0.50_Ga_0.50_P nanocrystals with increasing gallium content distribution in the ensemble.

To visualize changes to the relative intensity of the (002) peak more easily, we normalized the scattering vector at the (111) peak center and the (111) peak intensity in the simulated XRD patterns to 1 (Figure 4C). Performing the same normalization on the experimental data for the 6.25 nm InP nanocrystals (Figure 4E) shows that the (002) peak is considerably less intense than expected when compared to the reference intensity in red and the simulations for a pristine In_1–*x*_Ga_*x*_P nanocrystal (Figure 4C), suggesting we are not fully capturing the structure of our samples with the pristine zinc blende models. In Figure 4B, we calculated the powder diffraction patterns for an ensemble of In_1–*x*_Ga_*x*_P nanocrystals with included stacking faults and twin defects, which are well-known defects in these materials systems.^54^ We find that the intensity of the (002) peak decreases as planar defects are incorporated. The normalized (111) peak (Figure 4D) shows much better agreement with the experimental patterns, indicating our samples have considerable stacking disorder in the initial InP samples, and this disorder is maintained in the resulting In_1–*x*_Ga_*x*_P samples. These defects are also observable with HRTEM (Figure S10). The (200)/(111) peak intensity ratio can only be used to determine the gallium content in nanocrystals that have a pristine zinc blende lattice. The attenuation of the (200) peak in the experimental samples indicates that there is a considerable stacking fault density in these samples which may have important implications for their optoelectronic properties. Finally, we discuss several additional structural parameters including strain, shape, and inhomogeneous element distribution of In_1–*x*_Ga_*x*_P nanocrystals which PXRD is insensitive to in the Supporting Information (Figures S13–S15). Together these XRD simulations better refine our understanding of the structure of alloyed III–V colloidal nanocrystals.

The particle-to-particle compositional variation is another important source of heterogeneity we aim to understand. To do this, we first simulate the powder XRD patterns of a series of closely spaced particle compositions. Next, we weight the contribution of the scattering intensity for each composition by using a normal distribution to generate XRD patterns for an ensemble of particles with a given average composition and deviation. In Figure 4F, we show the simulated PXRD for In_0.50±σ_Ga_0.50±σ_P nanocrystals with composition standard deviations ranging from σ = 0.01 to σ = 0.20. We find that increasing the composition distribution causes a minute increase in the width of the diffraction peaks. This can be understood considering the composition-dependent lattice parameter leading to an increase in the peak width. However, this small effect is convoluted with the peak broadening due to Scherrer broadening, and in any experimental system even minuscule changes in crystallite size will almost certainly obscure any observable broadening related to composition distribution. Our simulations, therefore, demonstrate that PXRD alone is rather insensitive to compositional variations and provides important context for evaluating potential composition variation in many alloyed colloidal nanocrystal systems which have been synthesized and characterized by PXRD. Importantly, composition variations do not appear to affect the average composition measured by XRD for a given ensemble, indicating our measured diffusion coefficients represent averages for the ensemble. We attempt to directly investigate the gallium content distribution by measuring the lattice parameter from an ensemble of individual particles using HRTEM (Figures S16–S19) and find a small increase in lattice parameter variation with gallium incorporation. Unfortunately, at present, limitations in data collection and analysis requirements limit our current analysis to qualitative conclusions. We hope our initial investigations inspire future inquiry into compositional variation which may have important consequences for designing particles with narrow ensemble emission line widths.

In this work we have carefully studied several aspects of the InP→ In_1–*x*_Ga_*x*_P and InAs→ In_1–*x*_Ga_*x*_As cation exchange reactions on nanocrystals in molten KI/GaI_3_-based molten salts. We find that initially spherical InP nanocrystals are converted to faceted tetrahedron-shaped nanocrystals upon high-temperature annealing in molten salts. Furthermore, we measured the activation energy for gallium incorporation into InP and InAs nanocrystals and found it was much lower compared to related activation energies for bulk self-diffusion. We evaluated the structure of our In_1–*x*_Ga_*x*_P nanocrystals using powder XRD simulations and determined that these materials contain considerable stacking disorder present in the original InP nanocrystals which persist after the molten salt annealing. Together, our results highlight the substantial difference that exists between kinetic parameters pertaining to cation exchange in bulk and nanocrystalline III–V phases as well as the implications of cation exchange on the morphology of III–V nanocrystals.

## Supporting Information Available

Additional absorption spectra, X-ray diffraction patterns and corresponding peak shifts, SAXS data, photographs of the experimental setup, PXRD simulations to understand structural parameters of In_1–*x*_Ga_*x*_P nanocrystals, and HRTEM analysis of the gallium content distribution (PDF)

## References

[ref1] Moon H.; Lee C.; Lee W.; Kim J.; Chae H. Stability of Quantum Dots, Quantum Dot Films, and Quantum Dot Light-Emitting Diodes for Display Applications. Adv. Mater. 2019, 31 (34), 1804294 10.1002/adma.201804294. 30650209

[ref2] Shirasaki Y.; Supran G. J.; Bawendi M. G.; Bulović V. Emergence of colloidal quantum-dot light-emitting technologies. Nat. Photonics 2013, 7 (1), 13–23. 10.1038/nphoton.2012.328.

[ref3] Dai X.; Deng Y.; Peng X.; Jin Y. Quantum-Dot Light-Emitting Diodes for Large-Area Displays: Towards the Dawn of Commercialization. Adv. Mater. 2017, 29 (14), 1607022 10.1002/adma.201607022. 28256780

[ref4] Zhou J.; Pu C.; Jiao T.; Hou X.; Peng X. A Two-Step Synthetic Strategy toward Monodisperse Colloidal CdSe and CdSe/CdS Core/Shell Nanocrystals. J. Am. Chem. Soc. 2016, 138 (20), 6475–83. 10.1021/jacs.6b00674. 27144923

[ref5] Park Y. S.; Lim J.; Klimov V. I. Asymmetrically strained quantum dots with non-fluctuating single-dot emission spectra and subthermal room-temperature linewidths. Nat. Mater. 2019, 18 (3), 249–255. 10.1038/s41563-018-0254-7. 30617342

[ref6] Chen O.; Zhao J.; Chauhan V. P.; Cui J.; Wong C.; Harris D. K.; Wei H.; Han H.-S.; Fukumura D.; Jain R. K.; Bawendi M. G. Compact High-Quality CdSe–CdS Core–Shell Nanocrystals with Narrow Emission Linewidths and Suppressed Blinking. Nat. Mater. 2013, 12 (5), 445–451. 10.1038/nmat3539. 23377294 PMC3677691

[ref7] Das A.; Snee P. T. Synthetic Developments of Nontoxic Quantum Dots. ChemPhysChem 2016, 17 (5), 598–617. 10.1002/cphc.201500837. 26548450

[ref8] Del Alamo J. A. Nanometre-scale electronics with III–V compound semiconductors. Nature 2011, 479 (7373), 317–323. 10.1038/nature10677. 22094691

[ref9] Khan M. A.; Van Hove J. M.; Kuznia J. N.; Olson D. T. High electron mobility GaN/Al_x_Ga_1–x_N heterostructures grown by low-pressure metalorganic chemical vapor deposition. Appl. Phys. Lett. 1991, 58 (21), 2408–2410. 10.1063/1.104886.

[ref10] Davis R. F. III-V nitrides for electronic and optoelectronic applications. Proc. IEEE 1991, 79 (5), 702–712. 10.1109/5.90133.

[ref11] Jones A. C. Developments in metalorganic precursors for semiconductor growth from the vapour phase. Chem. Soc. Rev. 1997, 26 (2), 101 10.1039/cs9972600101.

[ref12] Allen P. M.; Walker B. J.; Bawendi M. G. Mechanistic insights into the formation of InP quantum dots. Angew. Chem., Int. Ed. 2010, 49 (4), 760–2. 10.1002/anie.200905632. PMC3119352 20025010

[ref13] Gary D. C.; Glassy B. A.; Cossairt B. M. Investigation of Indium Phosphide Quantum Dot Nucleation and Growth Utilizing Triarylsilylphosphine Precursors. Chem. Mater. 2014, 26 (4), 1734–1744. 10.1021/cm500102q.

[ref14] Franke D.; Harris D. K.; Xie L.; Jensen K. F.; Bawendi M. G. The Unexpected Influence of Precursor Conversion Rate in the Synthesis of III–V Quantum Dots. Angew. Chem., Int. Ed. 2015, 54 (48), 14299–14303. 10.1002/anie.201505972. 26437711

[ref15] Li Y.; Pu C.; Peng X. Surface activation of colloidal indium phosphide nanocrystals. Nano Res. 2017, 10 (3), 941–958. 10.1007/s12274-016-1353-x.

[ref16] Xie L.; Shen Y.; Franke D.; Sebastián V.; Bawendi M. G.; Jensen K. F. Characterization of Indium Phosphide Quantum Dot Growth Intermediates Using MALDI-TOF Mass Spectrometry. J. Am. Chem. Soc. 2016, 138 (41), 13469–13472. 10.1021/jacs.6b06468. 27690411

[ref17] Srivastava V.; Liu W.; Janke E. M.; Kamysbayev V.; Filatov A. S.; Sun C.-J.; Lee B.; Rajh T.; Schaller R. D.; Talapin D. V. Understanding and Curing Structural Defects in Colloidal GaAs Nanocrystals. Nano Lett. 2017, 17 (3), 2094–2101. 10.1021/acs.nanolett.7b00481. 28191964

[ref18] Lauth J.; Strupeit T.; Kornowski A.; Weller H. A Transmetalation Route for Colloidal GaAs Nanocrystals and Additional III–V Semiconductor Materials. Chem. Mater. 2013, 25 (8), 1377–1383. 10.1021/cm3019617.

[ref19] Tessier M. D.; Baquero E. A.; Dupont D.; Grigel V.; Bladt E.; Bals S.; Coppel Y.; Hens Z.; Nayral C.; Delpech F. Interfacial Oxidation and Photoluminescence of InP-Based Core/Shell Quantum Dots. Chem. Mater. 2018, 30 (19), 6877–6883. 10.1021/acs.chemmater.8b03117.

[ref20] Stein J. L.; Holden W. M.; Venkatesh A.; Mundy M. E.; Rossini A. J.; Seidler G. T.; Cossairt B. M. Probing Surface Defects of InP Quantum Dots Using Phosphorus Kα and Kβ X-ray Emission Spectroscopy. Chem. Mater. 2018, 30 (18), 6377–6388. 10.1021/acs.chemmater.8b02590.

[ref21] Won Y. H.; Cho O.; Kim T.; Chung D. Y.; Kim T.; Chung H.; Jang H.; Lee J.; Kim D.; Jang E. Highly efficient and stable InP/ZnSe/ZnS quantum dot light-emitting diodes. Nature 2019, 575 (7784), 634–638. 10.1038/s41586-019-1771-5. 31776489

[ref22] Ramasamy P.; Ko K.-J.; Kang J.-W.; Lee J.-S. Two-Step “Seed-Mediated” Synthetic Approach to Colloidal Indium Phosphide Quantum Dots with High-Purity Photo- and Electroluminescence. Chem. Mater. 2018, 30 (11), 3643–3647. 10.1021/acs.chemmater.8b02049.

[ref23] Kim Y.; Ham S.; Jang H.; Min J. H.; Chung H.; Lee J.; Kim D.; Jang E. Bright and Uniform Green Light Emitting InP/ZnSe/ZnS Quantum Dots for Wide Color Gamut Displays. ACS Appl. Nano Mater. 2019, 2 (3), 1496–1504. 10.1021/acsanm.8b02063.

[ref24] Zhao T.; Oh N.; Jishkariani D.; Zhang M.; Wang H.; Li N.; Lee J. D.; Zeng C.; Muduli M.; Choi H. J.; Su D.; Murray C. B.; Kagan C. R. General Synthetic Route to High-Quality Colloidal III-V Semiconductor Quantum Dots Based on Pnictogen Chlorides. J. Am. Chem. Soc. 2019, 141 (38), 15145–15152. 10.1021/jacs.9b06652. 31496238

[ref25] Srivastava V.; Janke E. M.; Diroll B. T.; Schaller R. D.; Talapin D. V. Facile, Economic and Size-Tunable Synthesis of Metal Arsenide Nanocrystals. Chem. Mater. 2016, 28 (18), 6797–6802. 10.1021/acs.chemmater.6b03501.

[ref26] Ginterseder M.; Franke D.; Perkinson C. F.; Wang L.; Hansen E. C.; Bawendi M. G. Scalable Synthesis of InAs Quantum Dots Mediated through Indium Redox Chemistry. J. Am. Chem. Soc. 2020, 142 (9), 4088–4092. 10.1021/jacs.9b12350. 32073841

[ref27] Liu W.; Chang A. Y.; Schaller R. D.; Talapin D. V. Colloidal InSb nanocrystals. J. Am. Chem. Soc. 2012, 134 (50), 20258–61. 10.1021/ja309821j. 23198950

[ref28] Srivastava V.; Kamysbayev V.; Hong L.; Dunietz E.; Klie R. F.; Talapin D. V. Colloidal Chemistry in Molten Salts: Synthesis of Luminescent In_1–x_Ga_x_P and In_1–x_Ga_x_As Quantum Dots. J. Am. Chem. Soc. 2018, 140 (38), 12144–12151. 10.1021/jacs.8b06971. 30125092

[ref29] Hudson M. H.; Gupta A.; Srivastava V.; Janke E. M.; Talapin D. V. Synthesis of In_1–x_Ga_x_P Quantum Dots in Lewis Basic Molten Salts: The Effects of Surface Chemistry, Reaction Conditions, and Molten Salt Composition. J. Phys. Chem. C 2022, 126 (3), 1564–1580. 10.1021/acs.jpcc.1c10394.

[ref30] Dirin D. N.; Dreyfuss S.; Bodnarchuk M. I.; Nedelcu G.; Papagiorgis P.; Itskos G.; Kovalenko M. V. Lead halide perovskites and other metal halide complexes as inorganic capping ligands for colloidal nanocrystals. J. Am. Chem. Soc. 2014, 136 (18), 6550–3. 10.1021/ja5006288. 24746226 PMC4524702

[ref31] Li Y.; Hou X.; Dai X.; Yao Z.; Lv L.; Jin Y.; Peng X. Stoichiometry-Controlled InP-Based Quantum Dots: Synthesis, Photoluminescence, and Electroluminescence. J. Am. Chem. Soc. 2019, 141 (16), 6448–6452. 10.1021/jacs.8b12908. 30964282

[ref32] Rusishvili M.; Wippermann S.; Talapin D. V.; Galli G. Stoichiometry of the Core Determines the Electronic Structure of Core–Shell III–V/II–VI Nanoparticles. Chem. Mater. 2020, 32 (22), 9798–9804. 10.1021/acs.chemmater.0c03939.

[ref33] Goldstein B. Diffusion in Compound Semiconductors. Phys. Rev. 1961, 121 (5), 1305–1311. 10.1103/PhysRev.121.1305.

[ref34] Fisher D. J. Diffusion in Semiconductors, Other Than Silicon: Compilation; Trans Tech Publications: Switzerland, 2010; pp 53–90.

[ref35] Wang L.; Wolk J. A.; Hsu L.; Haller E. E.; Erickson J. W.; Cardona M.; Ruf T.; Silveira J. P.; Briones F. Gallium self-diffusion in gallium phosphide. Appl. Phys. Lett. 1997, 70 (14), 1831–1833. 10.1063/1.118705.

[ref36] Langof L.; Ehrenfreund E.; Lifshitz E.; Micic O. I.; Nozik A. J. Continuous-Wave and Time-Resolved Optically Detected Magnetic Resonance Studies of Nonetched/Etched InP Nanocrystals. J. Phys. Chem. B 2002, 106 (7), 1606–1612. 10.1021/jp013720g.

[ref37] Mićić O. I.; Nozik A. J.; Lifshitz E.; Rajh T.; Poluektov O. G.; Thurnauer M. C. Electron and Hole Adducts Formed in Illuminated InP Colloidal Quantum Dots Studied by Electron Paramagnetic Resonance. J. Phys. Chem. B 2002, 106 (17), 4390–4395. 10.1021/jp014180q.

[ref38] Tessier M. D.; Dupont D.; De Nolf K.; De Roo J.; Hens Z. Economic and Size-Tunable Synthesis of InP/ZnE (E = S, Se) Colloidal Quantum Dots. Chem. Mater. 2015, 27 (13), 4893–4898. 10.1021/acs.chemmater.5b02138.

[ref39] Tessier M. D.; De Nolf K.; Dupont D.; Sinnaeve D.; De Roo J.; Hens Z. Aminophosphines: A Double Role in the Synthesis of Colloidal Indium Phosphide Quantum Dots. J. Am. Chem. Soc. 2016, 138 (18), 5923–9. 10.1021/jacs.6b01254. 27111735

[ref40] Zhang H.; Dasbiswas K.; Ludwig N. B.; Han G.; Lee B.; Vaikuntanathan S.; Talapin D. V. Stable colloids in molten inorganic salts. Nature 2017, 542 (7641), 328–331. 10.1038/nature21041. 28202966

[ref41] Kamysbayev V.; Srivastava V.; Ludwig N. B.; Borkiewicz O. J.; Zhang H.; Ilavsky J.; Lee B.; Chapman K. W.; Vaikuntanathan S.; Talapin D. V. Nanocrystals in Molten Salts and Ionic Liquids: Experimental Observation of Ionic Correlations Extending beyond the Debye Length. ACS Nano 2019, 13 (5), 5760–5770. 10.1021/acsnano.9b01292. 30964280

[ref42] Kim K.; Yoo D.; Choi H.; Tamang S.; Ko J.-H.; Kim S.; Kim Y.-H.; Jeong S. Halide–Amine Co-Passivated Indium Phosphide Colloidal Quantum Dots in Tetrahedral Shape. Angew. Chem., Int. Ed. 2016, 55 (11), 3714–3718. 10.1002/anie.201600289. 26849683

[ref43] Beberwyck B. J.; Surendranath Y.; Alivisatos A. P. Cation Exchange: A Versatile Tool for Nanomaterials Synthesis. J. Phys. Chem. C 2013, 117 (39), 19759–19770. 10.1021/jp405989z.

[ref44] Son D. H.; Hughes S. M.; Yin Y.; Paul Alivisatos A. Cation exchange reactions in ionic nanocrystals. Science 2004, 306 (5698), 1009–12. 10.1126/science.1103755. 15528440

[ref45] Li Z.; Saruyama M.; Asaka T.; Tatetsu Y.; Teranishi T. Determinants of crystal structure transformation of ionic nanocrystals in cation exchange reactions. Science 2021, 373 (6552), 332–337. 10.1126/science.abh2741. 34437152

[ref46] Le Bail A.; Duroy H.; Fourquet J. L. Ab-initio structure determination of LiSbWO_6_ by X-ray powder diffraction. Mater. Res. Bull. 1988, 23 (3), 447–452. 10.1016/0025-5408(88)90019-0.

[ref47] Crank J. The Mathematics of Diffusion, 2nd ed.; Oxford University Press: London, 1975; pp 89–103.

[ref48] Nelson A.; Honrao S.; Hennig R. G.; Robinson R. D. Nanocrystal Symmetry Breaking and Accelerated Solid-State Diffusion in the Lead–Cadmium Sulfide Cation Exchange system. Chem. Mater. 2019, 31 (3), 991–1005. 10.1021/acs.chemmater.8b04490.

[ref49] Sung Y.-M.; Lee Y.-J.; Park K.-S. Kinetic Analysis for Formation of Cd_1-x_Zn_x_Se Solid-Solution Nanocrystals. J. Am. Chem. Soc. 2006, 128 (28), 9002–9003. 10.1021/ja061858c. 16834351

[ref50] Shewmon P. Diffusion in Solids, 2nd ed.; Springer International Publishers: Switzerland, 2016; pp 189–222.

[ref51] Zhao J.; Chen B.; Wang F. Shedding Light on the Role of Misfit Strain in Controlling Core–Shell Nanocrystals. Adv. Mater. 2020, 32 (46), 2004142 10.1002/adma.202004142. 33051904

[ref52] Smith A. M.; Mohs A. M.; Nie S. Tuning the optical and electronic properties of colloidal nanocrystals by lattice strain. Nat. Nanotechnol. 2009, 4 (1), 56–63. 10.1038/nnano.2008.360. 19119284 PMC2711767

[ref53] Talapin D. V.; Mekis I.; Götzinger S.; Kornowski A.; Benson O.; Weller H. CdSe/CdS/ZnS and CdSe/ZnSe/ZnS Core–Shell–Shell Nanocrystals. J. Phys. Chem. B 2004, 108 (49), 18826–18831. 10.1021/jp046481g.

[ref54] Peng X.; Schlamp M. C.; Kadavanich A. V.; Alivisatos A. P. Epitaxial Growth of Highly Luminescent CdSe/CdS Core/Shell Nanocrystals with Photostability and Electronic Accessibility. J. Am. Chem. Soc. 1997, 119 (30), 7019–7029. 10.1021/ja970754m.

