# Supplementary material for: Diffusion-Limited Kinetics of Isovalent Cation Exchange in III–V Nanocrystals Dispersed in Molten Salt Reaction Media
Source: Nano Lett. 2022 Aug 11;22(16):6545–52. doi: 10.1021/acs.nanolett.2c01699 (PMC9413424; doi:10.1021/acs.nanolett.2c01699)
Supplement: Supplementary file 1 — nl2c01699_si_001.pdf [file nl2c01699_si_001.pdf]

Supplementary information

Diffusion-Limited Kinetics of Isovalent Cation Exchange in III-V Nanocrystals Dispersed in Molten Salt Reaction Media

Aritrajit Gupta,[†1] Justin C. Ondry,[†1] Min Chen,[†] Margaret H. Hudson,[†] Igor Coropceanu,[†] Nivedina A. Sarma,[†] and Dmitri V. Talapin[†‡]*

[†]*Department of Chemistry, James Franck Institute, and Pritzker School of Molecular Engineering, University of Chicago, Chicago, Illinois 60637, United States*

[‡]*Center for Nanoscale Materials, Argonne National Laboratory, Argonne, Illinois 60439, United States*

[1]These authors contributed equally.

*E-mail: dvtalapin@uchicago.edu

TABLE OF CONTENTS

Experimental section (Materials & Methods)	… S3-S9
Supplementary figures	… S10-S16
Discussion on XRD simulations	… S16-S21
HRTEM composition variation analysis	… S21-S26
Annexure: Diffusion in a sphere	… S27-S28
References	… S29-S30

EXPERIMENTAL SECTION

Chemicals: Trioctylphosphine (TOP, 97%), trioctylphosphine oxide (TOPO, 99%), and anhydrous solvents (hexane, toluene, ethanol (EtOH), isopropanol (IPA), acetonitrile (ACN)) were purchased from Sigma Aldrich and used as received. Oleylamine (OAm, technical grade, 70%) was purchased from Sigma Aldrich and purified by freezing, thawing, and then centrifuging to remove any insoluble solids. The resulting purified oleylamine was dried under dynamic vacuum at 100°C overnight and stored in a nitrogen glovebox. Oleic acid (OA, 90%) was purchased from Sigma Aldrich and dried under dynamic vacuum at 100°C overnight before storage in a nitrogen glove box. Potassium iodide (ultra-dry, 99.998%), gallium(iii) iodide (ultra-dry, 99.999%), hexamethylphosphorus triamide ($(NMe_2)_3P$, 97%), indium(i) chloride (99.995%) and N,N-dimethylformamide (DMF, anhydrous 99.9%) were purchased from Alfa Aesar and used as received. *Tris*(trimethylsilyl) phosphine ($(TMS)_3P$, 98%, stored frozen) *tris*(dimethylamino)arsine (99%), and indium(iii) chloride (anhydrous, 99.999%) were purchased from Strem Chemicals and used as received.

InP nanocrystal synthesis:

Small InP QDs. Small InP QDs (4 nm diameter) were obtained from Nanosys, Inc. as a solution in 1-octadecene and were stored in a nitrogen glove box. The QDs were precipitated by addition of EtOH and centrifugation, then redispersed in toluene for further processing.

Large, spherical InP QDs. Large, spherical InP QDs were synthesized by repeated applications of the procedure developed by Micic et al.[1-2] A portion of $InCl_3$ (1 g, 4.52 mmol) was dissolved in a solution of TOP (15 mL) and TOPO (1.5 g) at room temperature. This solution was filtered with a 0.2 µm PTFE syringe filter to remove undissolved solids. Half of this precursor mixture was stored separately, while the rest was loaded into a 100 mL 3-neck round-bottom flask under

nitrogen. A portion of $(TMS)_3P$ (375 mg, 2 mmol) was added to the $InCl_3$ solution, and the reaction was heated at 270 °C for 30 hours. The solution was cooled down to room temperature, another portion of $(TMS)_3P$ (375 mg, 2 mmol) was added to the precursor mixture stored previously and that was added to the reaction flask. It was heated at 280 °C for another 30 hours and finally cooled down. The QDs were washed in a nitrogen glove box three times by solvent/nonsolvent (toluene/EtOH) precipitation and centrifugation. As synthesized, these InP QDs are quite polydisperse, but the size distribution could be narrowed through size-selective precipitation. Fractions of successively smaller particles were isolated by slow addition of EtOH and centrifugation. The QDs were stored as a solution in toluene. The predominant fraction of the largest particles was used for this work.

Large, tetrahedral InP QDs. Large, tetrahedral InP QDs were synthesized based on an established procedure.[3-4] In a 100 mL 3-neck round bottom flask, 442 mg $InCl_3$ (2 mmol) was dissolved in 10 mL oleylamine and degassed under dynamic vacuum at 120 °C for an hour. The solution was heated to 260 °C under nitrogen, and 1.3 mL $(NMe_2)_3P$ (7.1 mmol) was quickly injected. The reaction proceeded at 260 °C for 30 minutes and was then cooled to room temperature naturally. The particles were washed three times by solvent/nonsolvent (toluene/IPA) precipitation and centrifugation. A narrower size distribution was achieved by size-selective precipitation, where EtOH nonsolvent was slowly added to a solution of QDs in toluene and successively smaller particles were isolated by centrifugation. The QDs were stored as a solution in toluene.

InAs nanocrystal synthesis: InAs nanocrystals were synthesized using InCl and *tris*(dimethylamino)arsine.[5] Briefly, 75ml of OAM was loaded into a 250ml European-style flask and degassed under dynamic vacuum at 110°C for several hours. The flask was cooled to room temperature, and 2.25g of InCl and 3ml of TOP were added under nitrogen flow. The reaction

mixture was heated to 240°C. Separately in a glovebox, 12ml OAM and 1.125ml of DMA-As were mixed and heated to 50°C until bubbles stopped forming. 4ml of this solution was loaded into a syringe and the remaining 8ml was loaded into a second syringe. The solution from the first syringe was injected into the InCl/OAM mixture at 240°C and the reaction was allowed to proceed for 20 min. Next, the remaining 8ml of OAM/DMA-As solution in the second syringe was injected at a rate of 4ml/hr. After the end of the injection, the reaction mixture was heated to 280°C for 20 min. Nanocrystals were recovered from the solution in a N_2 glovebox using anhydrous ethanol as a non-solvent and toluene as a solvent respectively.

Ligand exchange: Native organic ligands on the InAs and InP nanocrystals were replaced with GaI_3 using methods adapted from Dirin *et.al*.[6-7] Briefly in a N_2 glovebox, 5ml of 0.05M GaI_3 solution in DMF was prepared in a 20ml vial and 10ml of hexane was added to create a biphasic system. The nanocrystals dissolved in hexane were added to the top phase, and the biphasic mixture was stirred for several hours until phase transfer of the nanocrystals to the lower DMF phase was complete. The DMF phase was decanted and washed three times with excess hexane. Next, the nanocrystals were precipitated using ACN, collected by centrifugation, and dispersed again in DMF. The nanocrystals were then precipitated a second time using ACN and collected as a pellet *via* centrifugation. This pellet was then washed three times with excess ACN and dried overnight in a glovebox.

Molten salt dispersion: In a N_2 glovebox, GaI_3 and KI in either 1:1 or 65:35 molar ratio were loaded into an oven-dried mortar and pestle and ground into a fine powder. For InP nanocrystals, the previously prepared dry powder of ligand exchanged nanocrystals was added to the mortar and pestle with the eutectic mixture of GaI_3 and KI in 65:35 molar ratio and ground to disperse the powder in the salt mixture. Next the nanocrystal/salt mixtures were weighed out into oven dried

quartz ampoules (9mm outer diameter 7mm inner diameter, with an indentation ~50mm from the bottom), and a 6mm diameter quartz plug was inserted. The samples were heated at 240°C for 1 hour to melt the salt mixture. After melting, the ampoule was sealed using a H_2/O_2 torch under vacuum using an air free vacuum transfer chuck to mount the ampoule on a vacuum manifold without air exposure. The procedure was the same for InAs nanocrystals, except a KI/GaI_3 salt mixture in 1:1 molar ratio was used. It was melted together before adding the nanocrystals, allowed to solidify, and ground again into a fine power.

High temperature annealing: For InP samples, nanocrystals were annealed in a custom-built shaking furnace. The furnace consisted of ceramic walls around a central ceramic tube to hold the ampoule. The furnace was heated by a swaged heater cartridge with the temperature measured using a thermocouple and controlled using a PID temperature controller. The furnace assembly was placed atop a small vortex shaker. For InAs nanocrystals, the samples were annealed in a custom high temperature heating block, which consisted of a 4-inch diameter cylindrical aluminum block with 8, 3/8in diameter holes drilled in a circle around a central thermocouple hole. The block was wrapped in high temperature heating tape (BriskHeat BWH) and several layers of insulation. The temperature was controlled using a PID temperature controller. The temperature in each hole was independently measured to be within ±2°C of the set temperature.

Nanocrystal recovery from salt matrix. The ampoules were broken open in an N_2 glovebox, and the salt pellets embedded in the bottom of the ampoule were placed in an oven dried glass vial with a stir bar. Next, 5ml of ACN was added and stirred until the salt matrix dissolved completely. The nanocrystals were separated by centrifugation and the supernatant discarded. The nanocrystal pellet was washed with additional acetonitrile twice and recollected by centrifugation. For the $In_{1-x}Ga_xP$ samples, a solution of OAm and OA in toluene (50μl/ml each) was added to the pellet and

stirred until a clear colloidal solution was obtained. The $In_{1-x}Ga_xAs$ samples could be recovered as a colloidal solution in toluene using OAm alone (50µl/ml). The nanocrystals were then precipitated using anhydrous ethanol, followed by final dispersion in toluene.

Optical absorption. UV-vis spectra were collected on colloidal solutions of QDs with a Shimadzu UV-3600i Plus UV-Vis-NIR spectrophotometer in transmission mode.

Powder x-ray diffraction. Powder x-ray diffraction patterns were collected on a Rigaku MiniFlex x-ray diffractometer. Samples were deposited on (511) Si low background substrates. For $In_{1-x}Ga_xP$ nanocrystals, the lattice parameter was extracted by employing the Le Bail refinement framework to fit the full XRD pattern using the TOPAS software package (version 5, Bruker AXS). A twelfth-order Chebyshev polynomial was used to fit the background, and the crystalline phase was assumed to contain a single zinc blende phase (*F-43m* space group) exclusively. The lattice constants of bulk InP (5.8687 Å) and GaP (5.4505 Å) were used to calculate the alloy composition *x*, assuming a linear relationship between the lattice constant and alloying composition (Vegard's law) as follows:

$$a(In_{1-x}Ga_xP) = (1-x)a_{In} + xa_{Ga}$$

For $In_{1-x}Ga_xAs$ nanocrystals, the lattice parameter and crystallite size was determined by fitting the {111}, {220}, and {311} peaks to pseudo-Voigt functions to extract the peak width and position. Size was determined according to the Scherrer equation. The gallium composition was estimated by calculating the lattice parameter using the {111}, {220}, and {311} peaks and using a linear interpolation of the lattice parameters for the parent InAs and GaAs. Le Bail refinement and multipeak fitting result in nearly identical gallium content estimations.

Transmission electron microscopy. TEM images were obtained on an FEI Tecnai F30 microscope at 300 kV. For measurement of the gallium content distribution, special care was taken

to minimize microscope variations which would affect the magnification. All data was collected during the same TEM session. The illumination conditions were set such that the convergence angle of the illumination beam was identical for each sample by using a fixed “spot size” and C2 lens excitation. Next, the sample height was placed within 500 nm of eucentric focus using the stage height adjustment and all data was collected while maintaining the focus of the objective lens within ±500nm of the eucentric focus to minimize focus dependent magnification effects.

Small-angle x-ray scattering (SAXS). Colloidal solutions of QDs in toluene were prepared in sealed Kapton capillaries for small-angle x-ray scattering (SAXS) experiments. SAXS patterns were collected using a SAXSLab Ganesha instrument with Cu Kα radiation (λ = 1.54 Å). The SAXS curves were analyzed by fitting to a quantitative model in Igor Pro using the Irena package (available at http://usaxs.xray.aps.anl.gov/staff/ilavsky/irena.html).[8] The scattering curves were fit in the particle size distribution module using the model-free Maximum Entropy approach. Based on TEM data, the particles' form factor was assumed to be that of a sphere with an aspect ratio of 1. The extracted size distributions were further fit with symmetric Gaussians to estimate average particle diameters along with the associated standard deviations.

Powder x-ray diffraction simulations. Powder X-ray diffraction patterns were simulated from structures built using the Atomsk software package.[9] Briefly, large slabs of InP, GaP, and $In_{1-x}Ga_xP$ were generated by first preparing InP slabs, but with a lattice parameter which corresponded to the desired alloy gallium content. Next, a percentage of In atoms were randomly selected and converted to Ga atoms to generate alloy structures with the appropriate lattice constant and gallium composition. A spherical cutoff of desired diameter was used to generate nanocrystal structures. A similar approach was used to prepare $In_{1-x}Ga_xAs$ nanocrystal structures. Atomic structures were visualized using the VESTA software package.[10] X-ray diffraction patterns were simulated using

the DebyeByPy software package[11] which calculates intensities (I) as a function of the scattering vector (q) in units of $Å^{-1}$ using the Debye formula:

$$I(q) = \sum_{\mathrm{i}} \sum_{\mathrm{j}} f_{\mathrm{i}}(q) f_{\mathrm{j}}(q) \frac{\sin(q r_{\mathrm{ij}})}{r_{\mathrm{ij}}}$$

where the sums are over all of the atoms, f is the angle-dependent scattering factor calculated by Hartree-Fock wavefunctions by Cromer and Mann,[12] and r is the distance between atoms in Å.[13] Experimental data and simulated data was plotted either in q or in 2θ ($2\theta = 2\arcsin(\lambda q)$, where λ is wavelength of the X-ray source).

First-principles calculations initialization. For structural relaxation calculations, we determined the initial geometries of the nanoparticles studied here by cutting out different sized spherical nanoparticles from three dimensional zinc blende crystal structures using the Nanocut software.[14-15] To saturate the surface dangling bonds, fictitious hydrogen atoms with fractional charges were used. Each In atom is surrounded by four P(Ga) atoms, and the formal valence of the In atom is 3 and that of a P(Ga) atom is 5. Nominally, 0.75 electron of each In atom and 1.25 electron of each P(Ga) atom contribute to the formation of a In-P(Ga) covalent bond. In all the calculations, fictitious hydrogen atoms with charges of 0.75 electron and 1.25 electron were chosen to saturate In and P(Ga) atoms, respectively, so as to avoid the presence of any dangling bonds at the surfaces.

Geometry optimization. All geometry optimizations were performed at the DFT-PBE[16] level of theory with the Quantum Espresso package, using plane wave basis sets and norm-conserving pseudopotentials[17] with 60/240Ry for wavefunctions/charge energy cutoffs. Atomic positions were relaxed until the Hellmann-Feynman forces on each atom were smaller than 0.01 eV $Å^{-1}$.

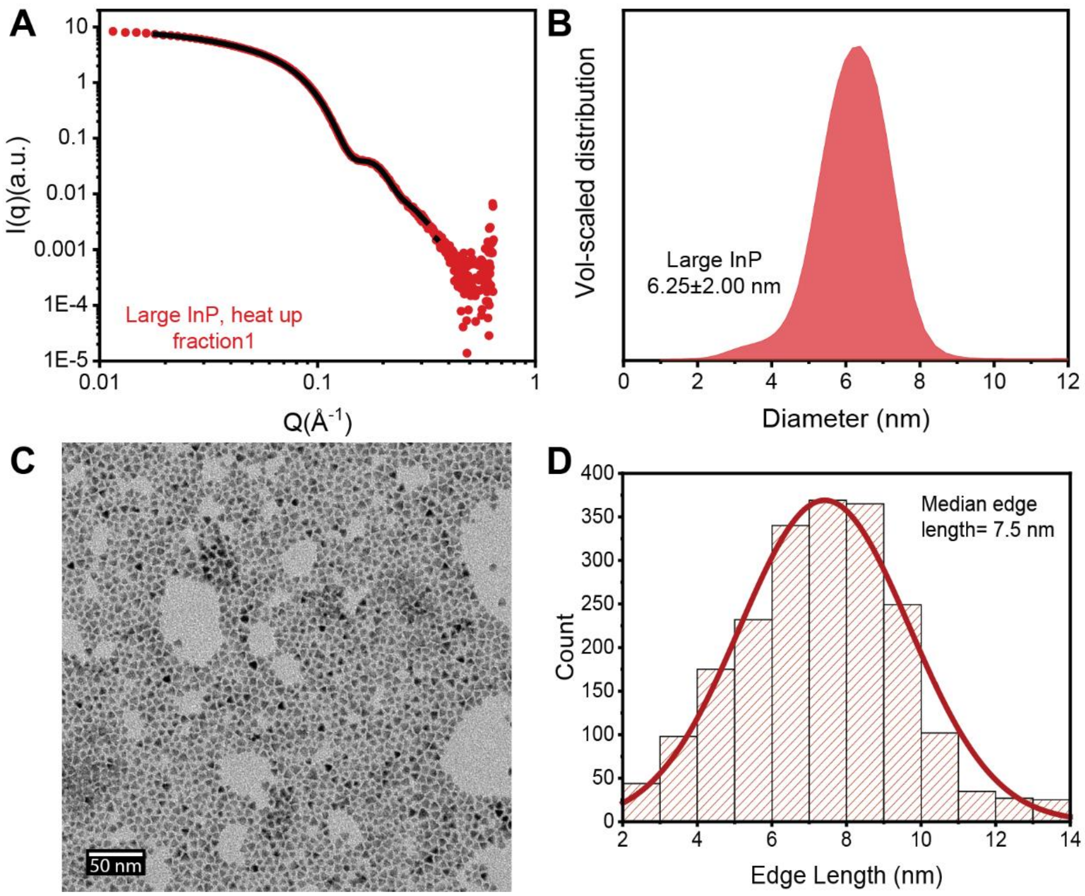

Figure S1. Size analysis. (A) Small-angle x-ray scattering (SAXS) data (red dots) and maximum-entropy fit (black line) for a solution of size-selected large spherical InP QDs. (B) Volume-scaled size distribution of the same obtained from a maximum-entropy fit to the SAXS data. (C) TEM of size-selected large tetrahedral InP QDs, and (D) the corresponding size analysis. The triangular projections on TEM were used to estimate an average edge length.

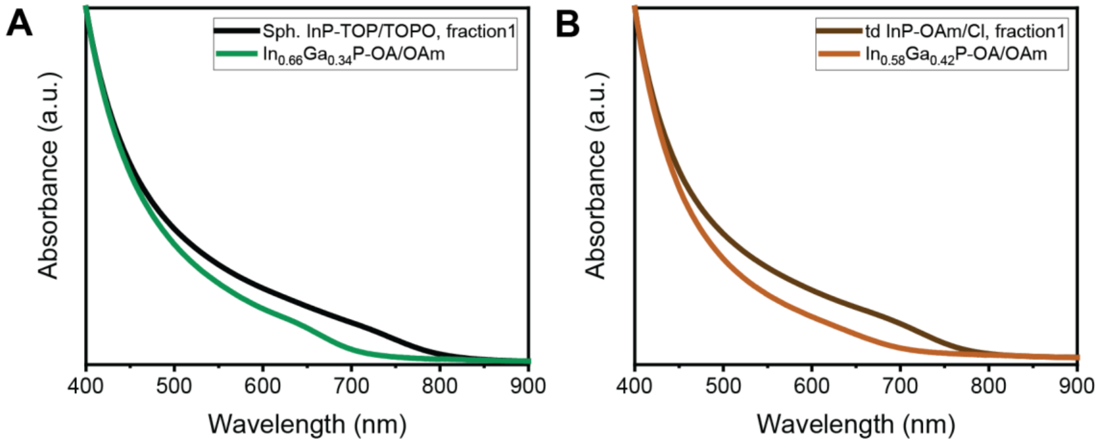

Figure S2. A blue shift of the absorption onset observed after In^{3+} to Ga^{3+} cation exchange was performed on (A) large spherical InP QDs over an annealing duration of 28 min at 393°C and (B) large tetrahedral InP QDs over an annealing duration of 60 min at 440°C in a eutectic iodide molten salt medium.

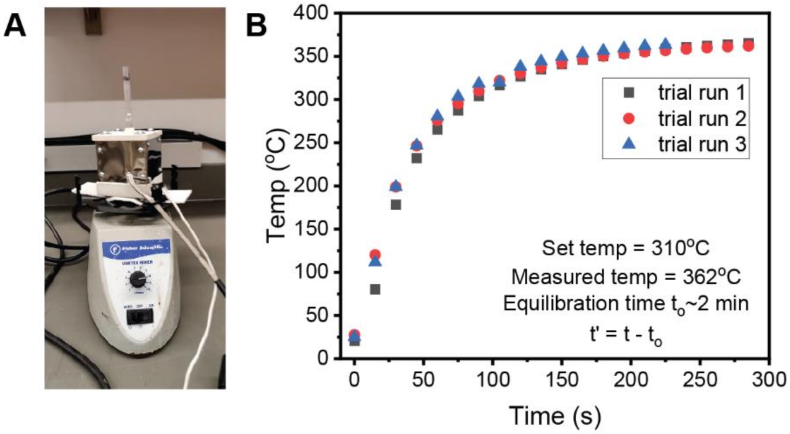

Figure S3. (A) A custom-built shaking furnace setup used for high temperature annealing of InP nanocrystals. (B) The temperature response curve of this custom-built furnace was constructed using an independent temperature probe. Based on this behavior, a temperature equilibration period of 2 min was subtracted from the recorded annealing durations.

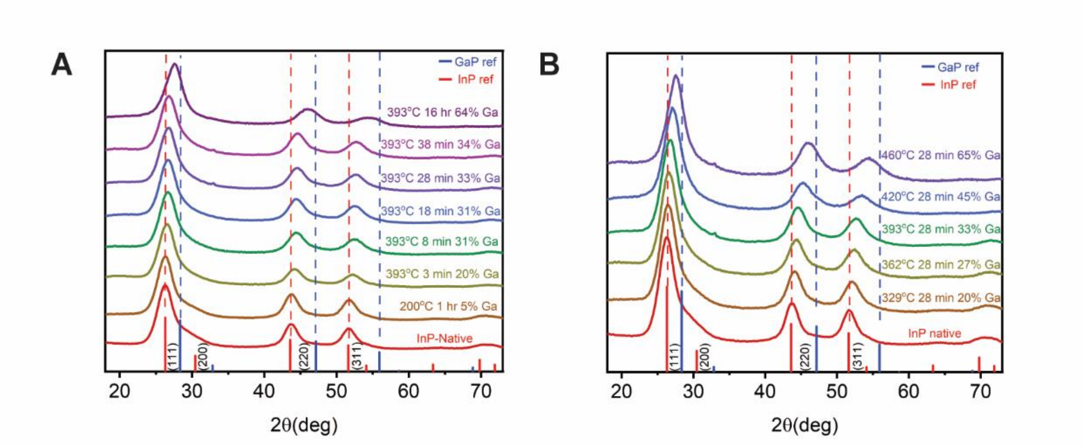

Figure S4. Powder XRD patterns (in the conventional 2θ axis for reference) for ~6.25 nm InP nanocrystals annealed in GaI_3/KI [65:35 mol%] for different times (A) and temperatures (B).

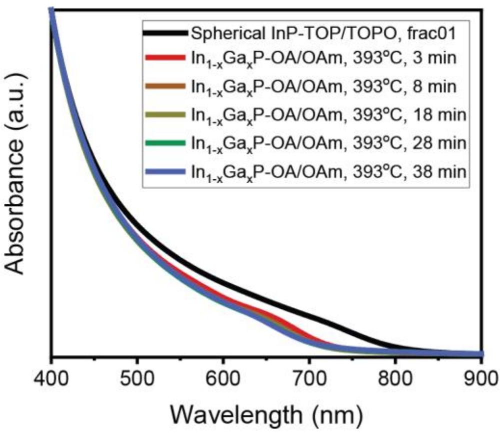

Figure S5. Absorption spectra of the ~6.25 nm InP particles, along with the $In_{1-x}Ga_xP$ populations synthesized from that via In^{3+} to Ga^{3+} cation exchange reaction performed in a molten GaI_3/KI eutectic salt mixture. The nanocrystals were annealed at 393°C for different durations, and the alloyed particles were recovered as a colloidal solution using a 1:1 mixture of oleylamine and oleic acid ligands.

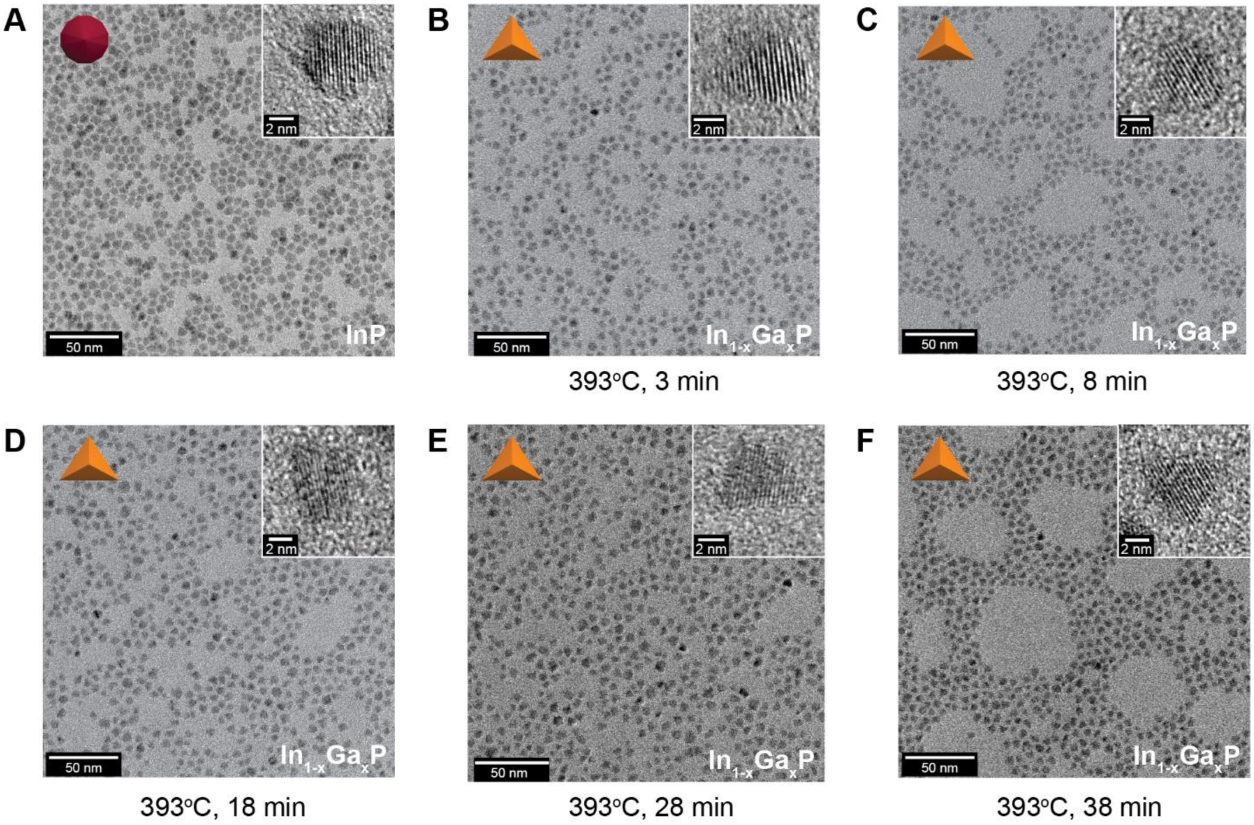

Figure S6. TEM images of the spherical ~6.25 nm InP nanocrystals (A) before and (B-F) after annealing in the GaI_3/KI eutectic molten salt reaction medium over different durations. The cation exchanged particles appear increasingly more faceted, characterized by triangular projections on TEM indicating tetrahedral morphology.

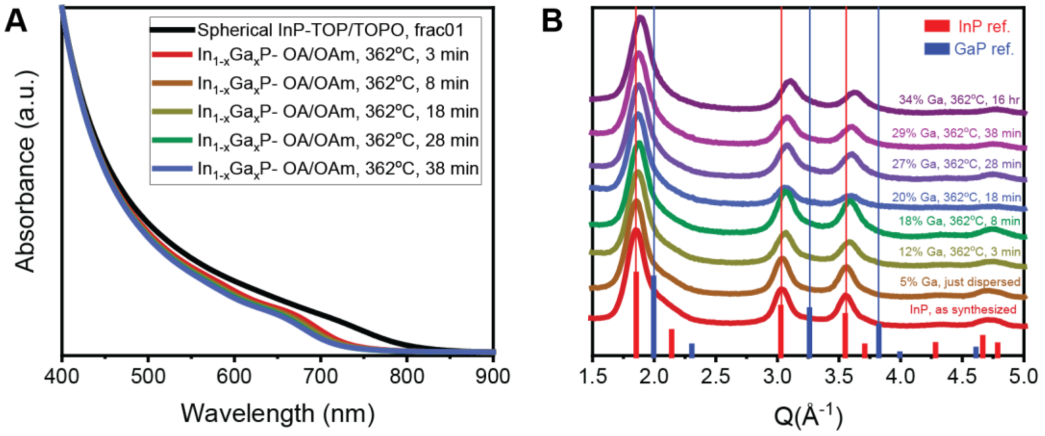

Figure S7. (A) Absorption spectra of the size-selected large spherical InP particles, along with the $In_{1-x}Ga_xP$ populations synthesized by annealing them in the molten salt reaction medium at 362°C for different durations. The cation exchanged particles were recovered as a colloidal solution using a 1:1 mixture of oleylamine and oleic acid ligands. (B) The corresponding diffraction patterns indicate the presence of a pure zinc blende phase. The lattice constants were estimated with Le Bail refinement, and subsequently the Ga compositions were calculated using Vegard's law.

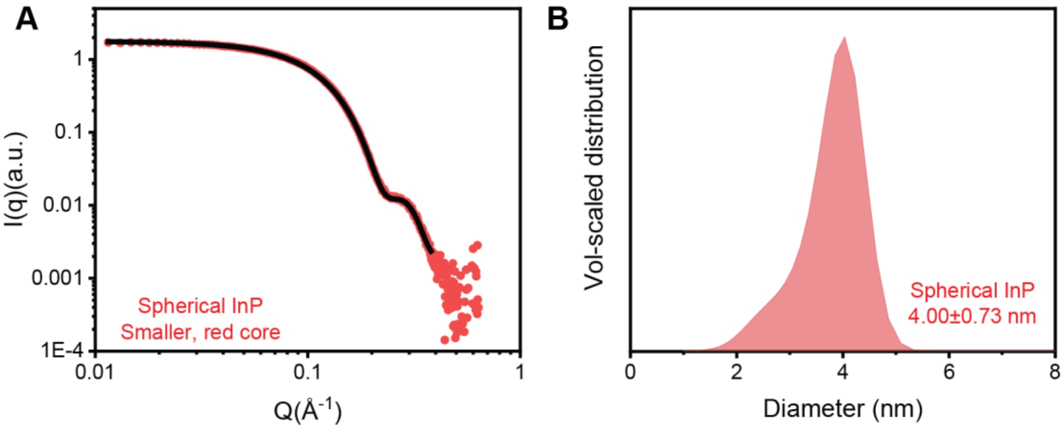

Figure S8. (A) Small-angle x-ray scattering (SAXS) data (red dots) and maximum-entropy fit (black line) for a solution of small spherical InP QDs obtained from Nanosys. (B) Volume-scaled size distribution of the same obtained from a maximum-entropy fit to the SAXS data.

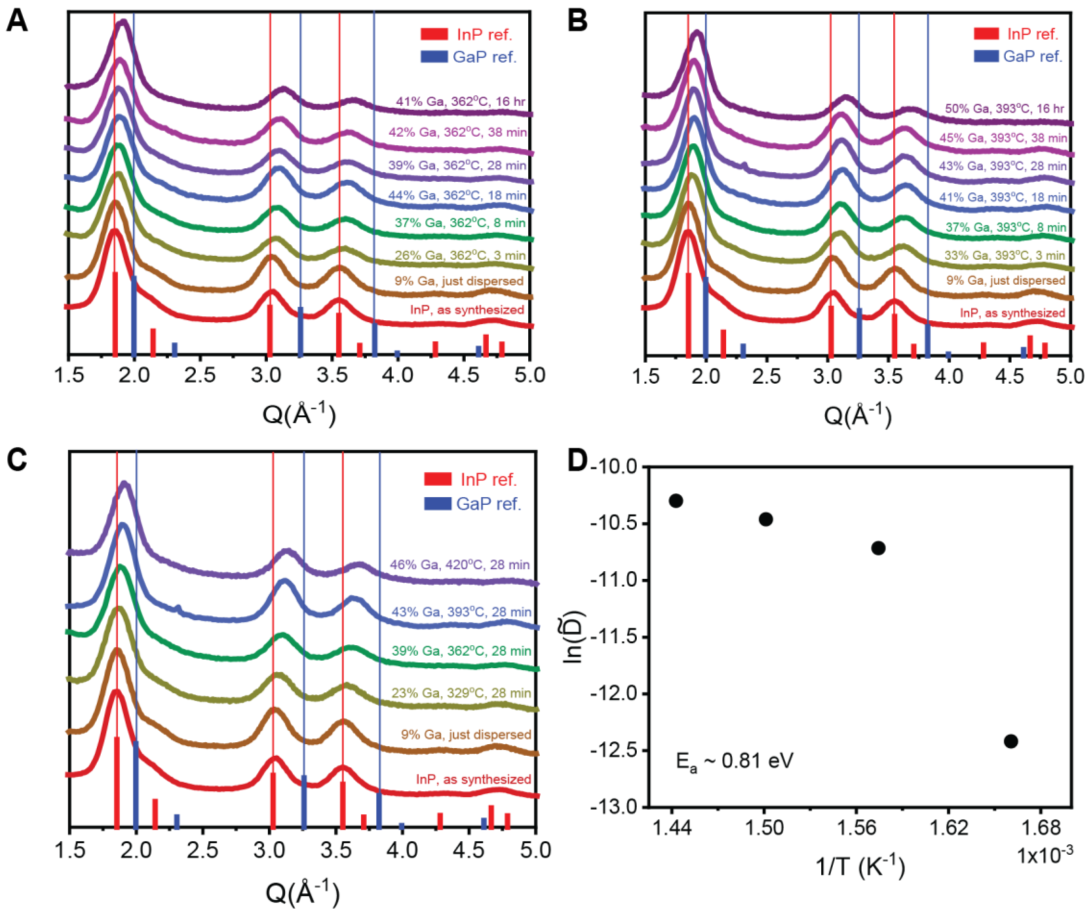

Figure S9. Powder XRD patterns for ~4 nm InP nanocrystals annealed in GaI_3/KI [65:35 mol%] for different times at (A) 362°C and (B) 393°C. (C) Diffraction patterns were also recorded for particles annealed at different temperatures. The gallium content extracted from the temperature series data, was used to estimate an apparent diffusion coefficient at each temperature. (D) Arrhenius plot of the apparent diffusion coefficient measured as a function of temperature. The fit deviates from linearity significantly, rendering the extracted activation energy questionable.

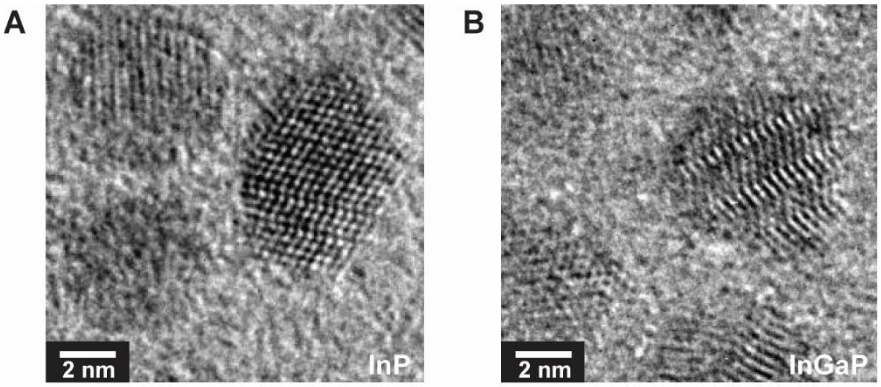

Figure S10. High-resolution TEM images confirm the frequent presence of planar defects such as twin planes and stacking faults in the (A) as synthesized large spherical InP nanocrystals and (B) alloyed $In_{1-x}Ga_xP$ nanocrystals synthesized by annealing InP in a GaI_3/KI eutectic molten salt at 393°C for 38 min.

Supplementary Discussion on XRD simulations

We aim to understand how other structural changes (*e.g.,* shape, core/shell structure, *etc.*) influence the expected PXRD patterns for $In_{1-x}Ga_xP$ nanocrystals. We observe that the (002) peak intensity is modulated by the particle shape. Tetrahedron-shaped particles give a broader (002) peak compared to spherical particles of the same volume; however, the integrated area of the peak appears unaffected by the shape (Figure S12). This indicates that stacking disorder rather than the observed shape changes is likely the main factor attenuating the (002) peak intensity in our nanocrystal samples. Finally, we consider the partitioning of the gallium and indium atoms in the nanocrystals. We simulated PXRD patterns for $In_{0.50}Ga_{0.50}P$ structures with random alloy and core/shell geometries (Figure S13). Here we find that the calculated XRD pattern is minimally affected by the distribution of the gallium and indium, with only miniscule changes to the width of the (002) peak for inhomogeneous elemental distributions. Further, accounting for strain in the

core shell structure leads to minimal changes to the observed PXRD pattern (Figure S14). Based on comparison of our experimental PXRD patterns and the simulations, we can conclude that our $In_{1-x}Ga_xP$ nanocrystals have considerable stacking disorder, and that gallium is incorporated based on the peak shifts, but PXRD does not provide adequate observing power to discern minute changes to the particle shape or the elemental distribution within a single nanocrystal. More advanced X-ray or TEM based techniques will likely be needed to understand these structural parameters.

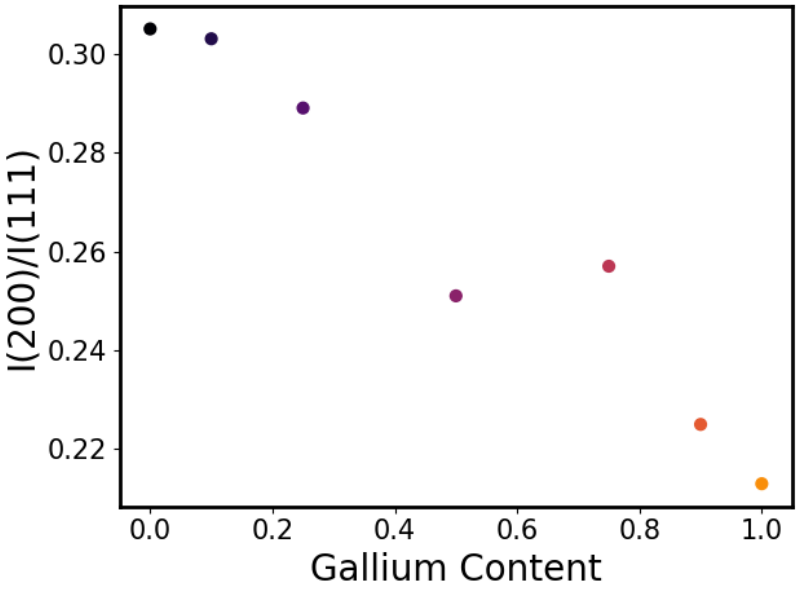

Figure S11. Ratio of the intensity of the (200) and the (111) peaks for $In_{1-x}Ga_xP$ nanocrystals with different gallium content showing a strong trend for decrease in the relative intensity of the (200) peak as gallium content increases.

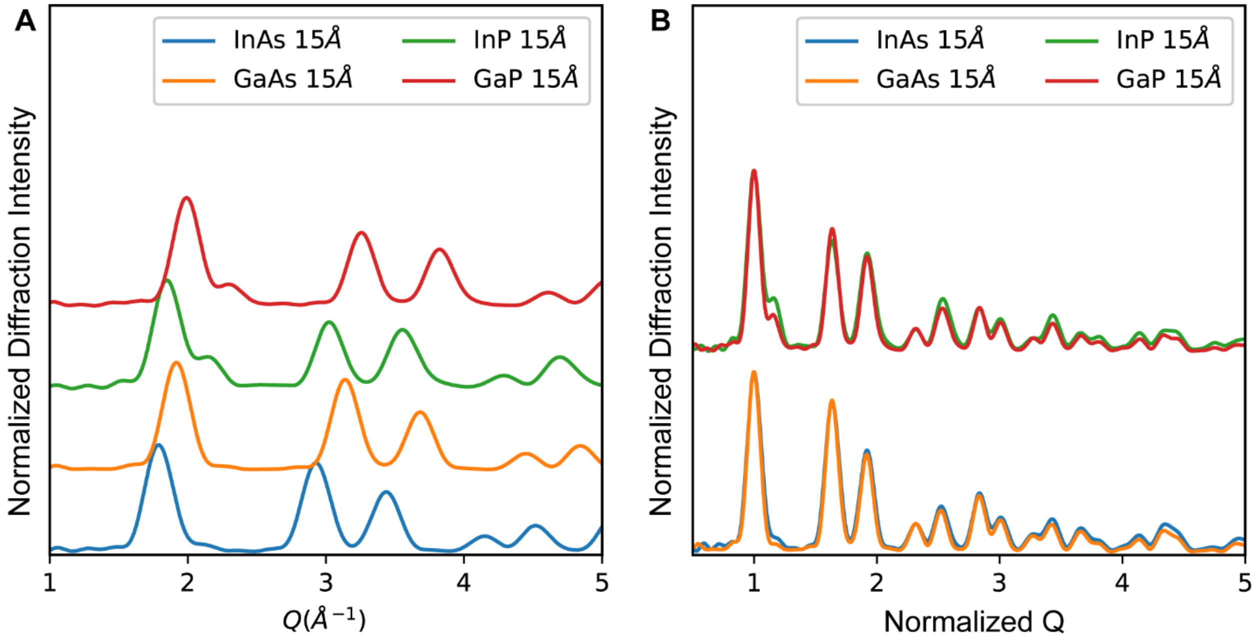

Figure S12. (A) Powder XRD simulations of InAs, GaAs, InP, and GaP nanocrystals respectively showing the smaller magnitude of change in the intensity of the (200) peak with changing composition and (B) plotted by normalizing the scattering vector to the (111) position and intensity to facilitate comparison.

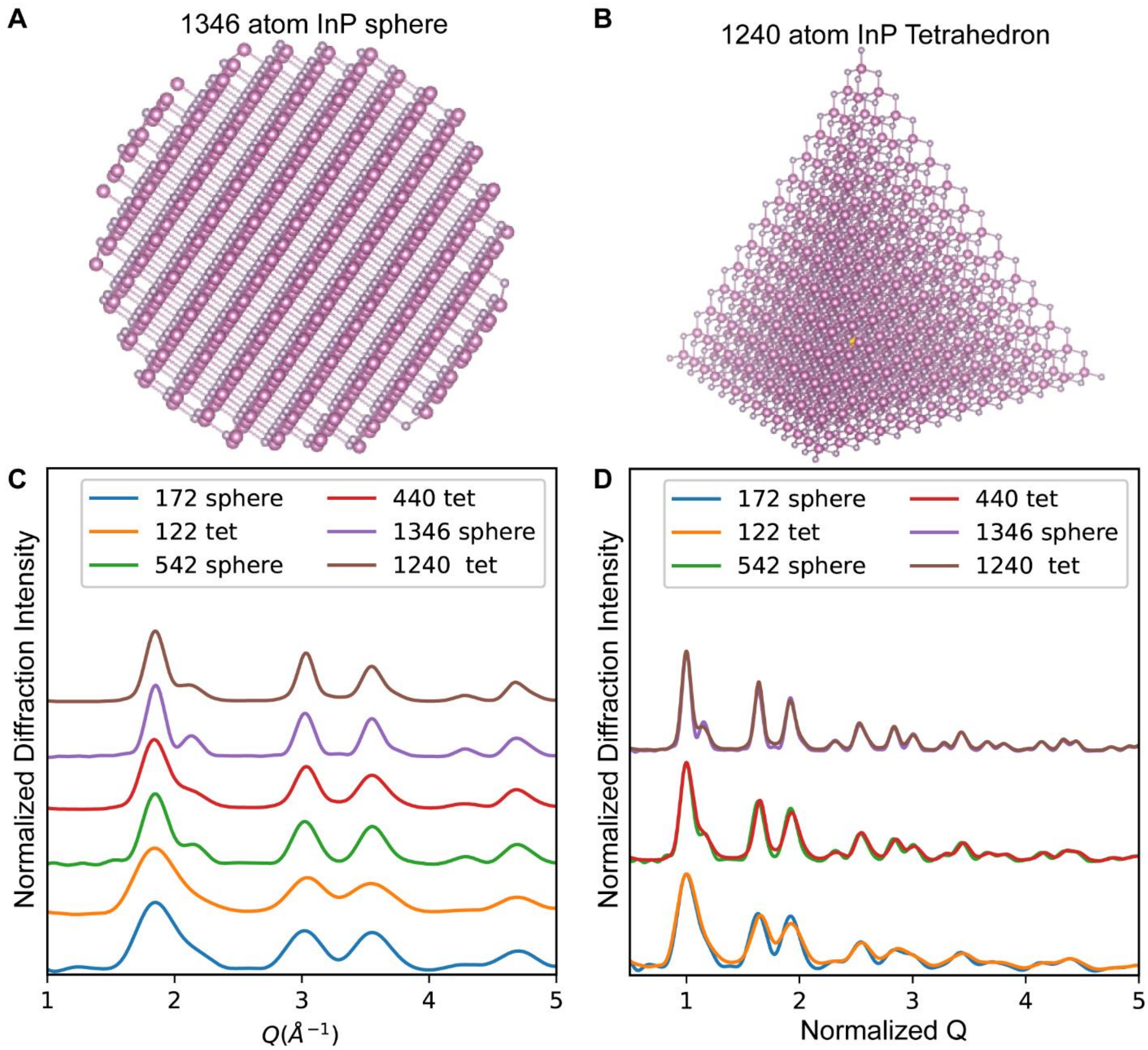

Figure S13. Effect of shape on PXRD patterns of InP nanocrystals. Example of a nearly equal volume (A) sphere-and (B) tetrahedron-shaped InP nanocrystal. (C) Simulated powder XRD patterns of sphere-and tetrahedron- shaped InP nanocrystals of different sizes. (D) Comparison of the PXRD patterns with normalized intensity and scattering vector for nearly identical volume sphere- and tetrahedron-shaped InP nanocrystals.

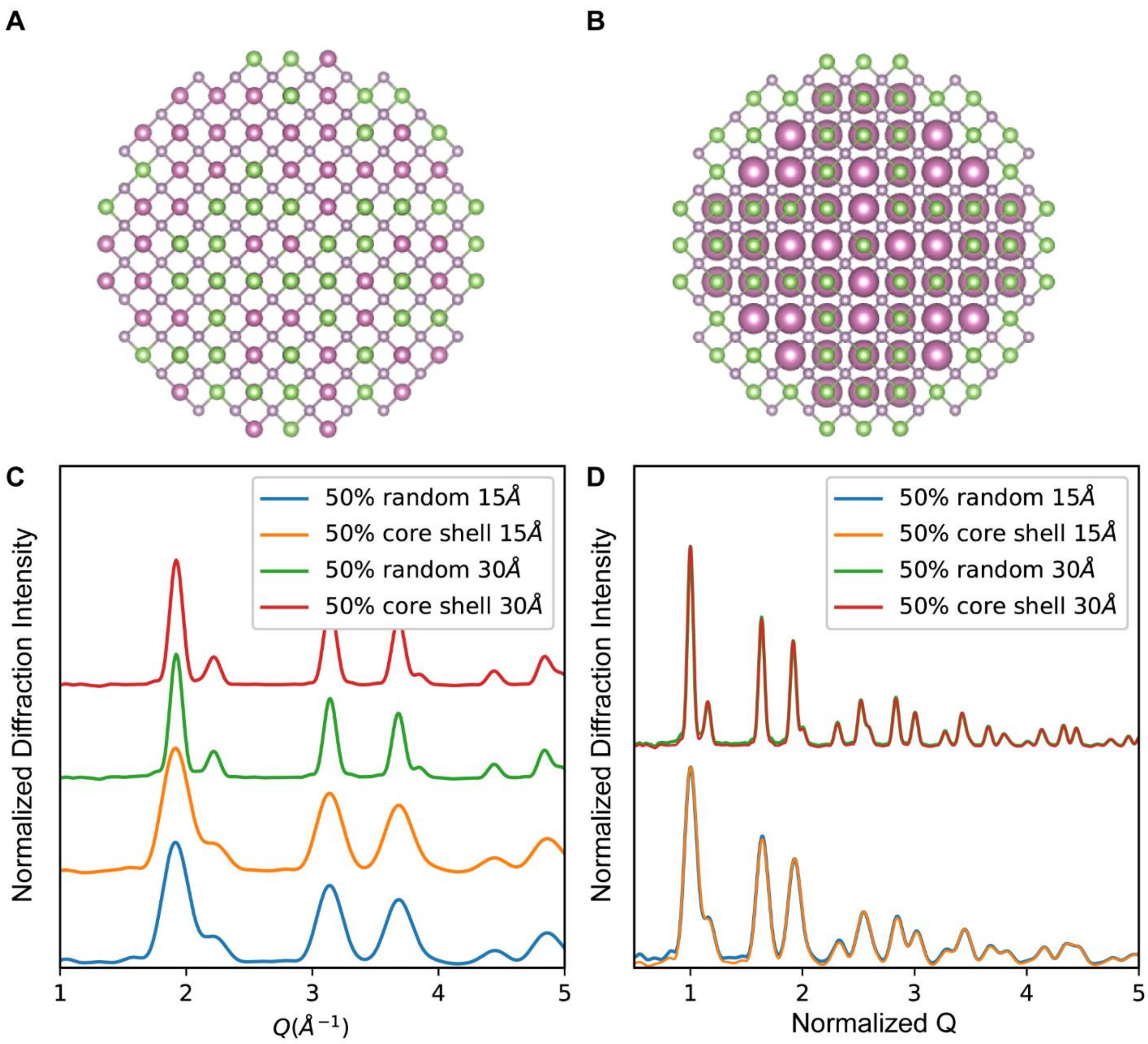

Figure S14. Effect of elemental distribution on PXRD patterns of $In_{1-x}Ga_xP$ nanocrystals. Example structure of a random alloy of $In_{0.50}Ga_{0.50}P$ (A) and a core shell structure with an overall composition of $In_{0.50}Ga_{0.50}P$. (B), (C) PXRD simulations of two different sized random alloy $In_{0.50}Ga_{0.50}P$ and core/shell $In_{0.50}Ga_{0.50}P$ structures. (D) Comparison of the PXRD patterns with normalized intensity and scattering vector for the 15Å and 30Å radius particles.

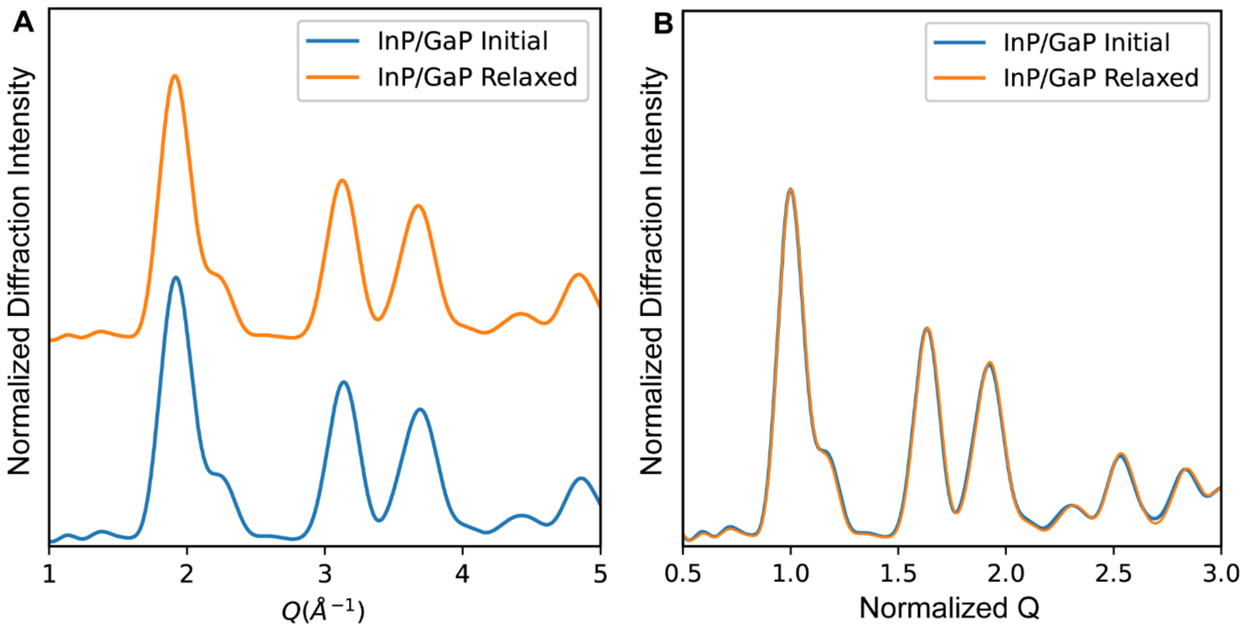

Figure S15. Simulated PXRD patterns for (A) an $In_{1-x}Ga_xP$ structure before DFT relaxation (blue) and after a structural minimization routine (orange). (B) Overlay of the structures to highlight changes in the peak shapes or relative intensities due to the strain relaxation.

HRTEM Composition Variation Analysis

Elucidating the distribution of gallium content among an ensemble of $In_{1-x}Ga_xP$ nanocrystals remains an important problem. To this end, we collect HRTEM images of InP and $In_{1-x}Ga_xP$ nanocrystals using identical imaging conditions (see methods). From these we calculate the lattice constant for ~120 individual InP and $In_{1-x}Ga_xP$ nanocrystals using an automated MATLAB routine (see methods and Figure S15). A probability density histogram of the measured lattice parameters for an ensemble of individual InP nanocrystals is shown in Figure S18A. A Gaussian fit to the histogram returns a distribution centered at 5.89Å, in good agreement with bulk InP, and a standard deviation of 0.057Å. The standard deviation of the lattice parameter measurement for the InP nanocrystals (which should have nearly identical lattice constants) represents a measure of the

precision of the HRTEM measurement approach we have used. We can thus ascribe any increase in the measured distribution width to real increases in the measured lattice parameter variation. We note that microscope focus variations and tilting the crystals off zone axis (which could vary sample-to-sample) appear to have negligible effects on the measured lattice parameter for these nanocrystals as suggested by multislice HRTEM image simulations (Figure S16). We performed identical measurements on a sample of $In_{1-x}Ga_xP$ nanocrystals and found a lattice constant distribution centered at 5.76Å with a standard deviation of 0.088Å (Figure S18B). This indicates there is a measurable increase in the width of the lattice constant distribution upon incorporation of Ga into InP nanocrystals.

One possible explanation for observed broadening is that the size dependent kinetics of gallium incorporation observed in the previous section cause the smaller nanocrystals in the ensemble to incorporate more gallium than the larger nanocrystals in the ensemble. As a result, we would expect to observe a correlation between particle size and measured lattice parameter where smaller particles have a smaller lattice parameter. To this end, we developed an automated approach to measure the size of each particle using HRTEM (Figure S17). For InP we observe a weak positive correlation between particle size and measured lattice constant. Upon incorporation of Ga, we observe the slope of this correlation meaningfully increases, indicating the gallium incorporation is nonuniform among the $In_{1-x}Ga_xP$ particles depending on their size.

This points out an important practical consideration for preparing $In_{1-x}Ga_xP$ nanocrystals with narrow emission linewidths. First, starting with an InP sample with narrow size distribution is critical, since disproportionate incorporation of gallium into the smaller particles serves to blue shift their emission, which is already on the blue edge of the emission spectrum. Second, reaction conditions must be developed which minimize size dependent gallium incorporation. For example,

developing cation exchange conditions through which the extent of gallium incorporation is determined by thermodynamics rather than through diffusion kinetics would allow for a steady state condition to be achieved, thus minimizing size dependent gallium incorporation. This endeavor likely will need greater precision for the single particle lattice parameter measurement, which will be possible using modern highly stable TEMs and appropriate additional data analysis considerations.[18] For example in our analysis we used any particle which displayed (111) lattice fringes for determining the lattice parameter. In previous work using this approach on metal nanocrystals,[18] a key data analysis requirement was to only measure particles which were on a well defined zone axis such that the crystal tilt could be precisely known and thus even small errors in lattice parameter determination from crystal tilt could be avoided. At present it was not feasible to collect enough data such that reasonable statistics of particles on zone axis could be obtained. Advances in automated TEM data collection may enable this in near future.

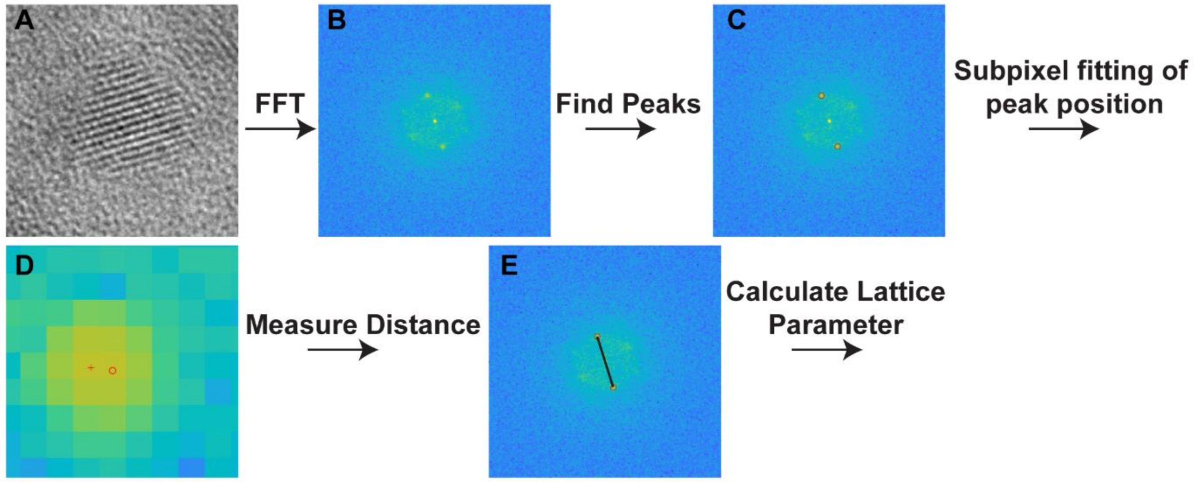

Figure S16. Routine for measuring the lattice constant for an ensemble of individual InP and $In_{1-x}Ga_xP$ nanocrystals. (A) a single nanocrystal is cropped from a larger image, next (B) a fast Fourier transform of the image is calculated (with a Hanning window to avoid artifacts). The two opposing

(111) peaks are identified using the fast peak find algorithm[19] in MATLAB. Next, the peak positions were refined by fitting the peak position with subpixel accuracy (D) by using a Taylor expansion of a 3 by 3 window around the maximum intensity pixels and determining where the derivative would be zero. The (111) spacing was measured as the distance between the two (111) reflections in the FFT. The lattice parameter was calculated assuming the particles had an ideal cubic zinc blende phase. Only particles which had (111) planes as the most intense peaks were used. Particles rarely had more intense (100) or (110) peaks, and in these cases appropriate distance cutoff filters were applied to remove those cases.

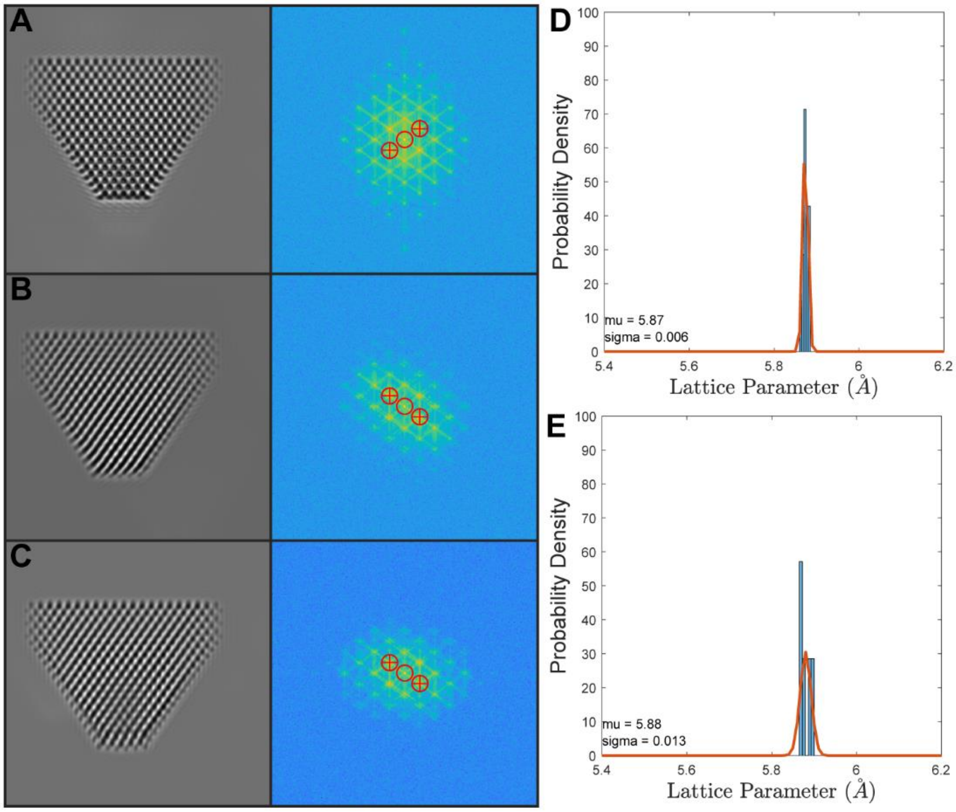

Figure S17. Example multislice HRTEM image simulations of an InP tetrahedron (A) perfectly on the (110) zone axis, (B) tilted 4° around the X and 4° around the Y axis, and (C) tilted 6° around the X and 2° around the Y axis with their associated Fourier transforms and fitted peak positions. (D) Histogram of the measured lattice parameter for 15 tilt conditions ranging from 0° around the

X and Y axes, and 10° around both X and Y axes simultaneously. (E) Histogram of the measured lattice parameter of 6 defocus conditions for a particle tilted off the (110) by 4° in X and Y. Images were simulated using the Computem software package (https://sourceforge.net/projects/computem/). Imaging parameters for the simulated images were set for near Scherzer conditions based on estimated parameters for our Tecnai F30 S-Twin TEM. Simulation parameters are as follows: Accelerating voltage 300keV, C3 Spherical aberration coefficient: 1.2mm, Defocus: -58nm, Δ Defocus: 10nm, Beam convergence angle: 0.5mrad, Objective aperture: 20mrad, transmission function size: 512 by 512-pixels and slice thickness: 2Å. All higher order aberrations were set to be zero since our instrument is C3 limited and all higher order aberrations are insignificant compared to C3. Images were simulated at 0K (no atom vibrations).

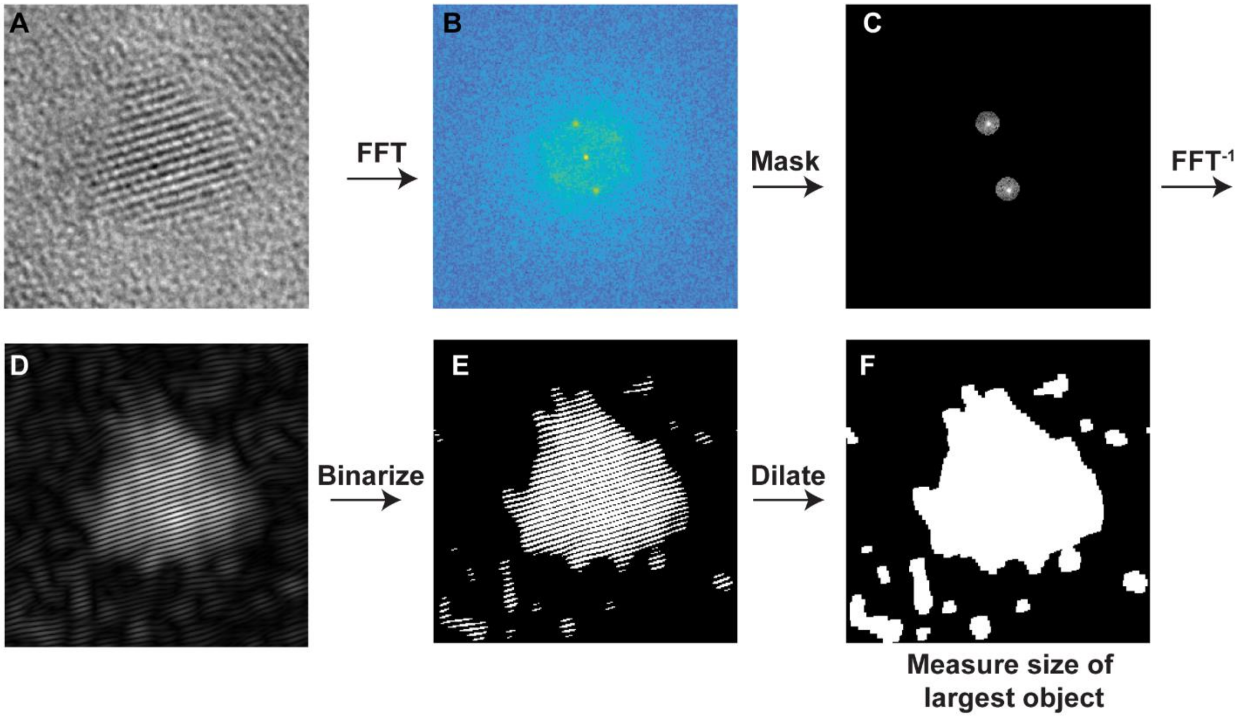

Figure S18. Routine for determining the particle size from HRTEM images. (A) Initial HRTEM image of an InP nanocrystals followed by computing the FFT of the image (B). Next a mask is

applied to isolate the signal from the (111) lattice fringes (C) followed by computing the inverse FFT (D). Next the Fourier filtered image is binarized (E) with a threshold which only keeps the (111) lattice fringes. Finally, this image is dilated to fill in the dark space between the lattice fringes (F) and the area of the particle is determined. We quantify particle size as the diameter of a circle which would have equal area to the measured irregular region. We note that this measurement is prone to several sources of error. First, the quality of the lattice fringes is different image-to-image, and thus it is difficult to reliably define an intensity cutoff for the binarization step. For these reasons, there is a large spread in the area determinations compared to the size distribution of the sample measured by conventional TEM methods; however, on average particles which are larger give larger areas as determined by the automated method used here. For these reasons, we say our results are semi-quantitative and serve to illustrate the principle of size dependent gallium incorporation but may not provide a precise or accurate measure of the degree of this effect.

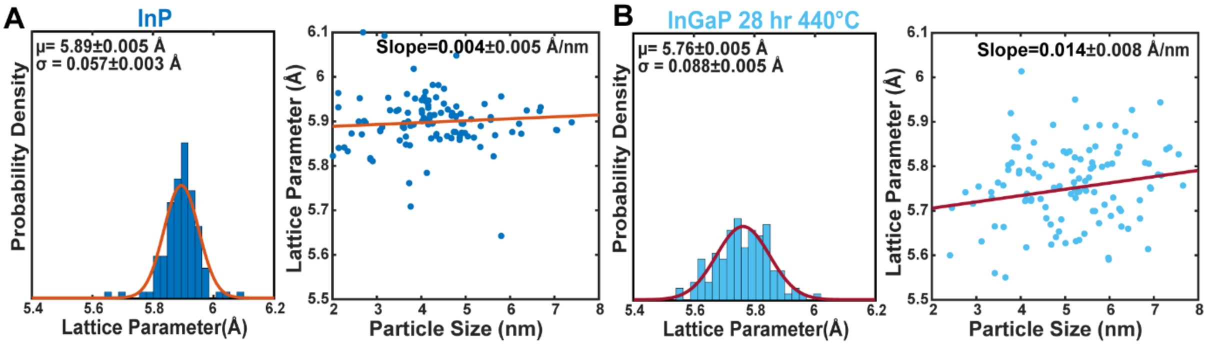

Figure S19. (A) Measured lattice constant distribution for InP nanocrystals determined via single particle HRTEM measurements (left) and the correlation between particle size and lattice constant (right). (B) Measured lattice constant distribution for $In_{1-x}Ga_xP$ nanocrystals determined via single particle HRTEM measurements (left) and the correlation between particle size and lattice constant (right).

ANNEXURE

Diffusion in a Sphere: Non-steady state with a constant surface concentration[20]

The mathematical theory of diffusion in isotropic substances is based on the hypothesis that the rate of transfer of diffusing substance through unit area of a section is proportional to the concentration gradient measured normal to the section, i.e.

$$F = -D\ \partial C/\partial x \qquad \text{... (1)}$$

where F is the rate of transfer per unit area of section, C the concentration of diffusing substance, x the space coordinate measured normal to the section, and D is called the diffusion coefficient. If the diffusion coefficient is constant, the rate of diffusion reduces to

$$\frac{\partial C}{\partial t} = D\frac{\partial^2 C}{\partial x^2} \qquad \text{... (2)}$$

provided the gradient of concentration is only along the x-axis. These equations are referred to as Fick's first and second laws of diffusion. If the surface concentrations are constant with an initial distribution $f(x)$, *i.e.*

$$C = C_1,\ \ x = 0,\ \ t \geq 0;$$

$$C = C_2,\ \ x = l,\ \ t \geq 0;$$

$$C = f(x),\ \ 0 < x < l,\ \ t = 0;$$

The general solution in the form of a trigonometric series is

$$C = C_1 + (C_2 - C_1)\frac{x}{l} + \frac{2}{\pi}\sum_{1}^{\infty}\frac{C_2\cos(n\pi - C_1)}{n}\sin\left(\frac{n\pi x}{l}\right)\exp(-Dn^2\pi^2 t/l^2) + \frac{2}{l}\sum_{1}^{\infty}\sin\left(\frac{n\pi x}{l}\right)\exp(-Dn^2\pi^2 t/l^2)\int_0^l f(x')\sin\left(\frac{n\pi x'}{l}\right)dx' \qquad \text{... (3)}$$

In most cases $f(x)$ is either zero or constant; therefore, the integral in equation (3) can be evaluated readily. Now, consider a spherical particle with radial coordinate r. When the diffusion is only in the radial direction, the diffusion equation (2) for a constant diffusion coefficient takes the form

$$\frac{\partial C}{\partial t} = \widetilde{D}(\frac{\partial^2 C}{\partial r^2} + \frac{2}{r}\frac{\partial C}{\partial r}) \qquad \text{... (4)}$$

Substituting $u = Cr$, (4) becomes

$$\frac{\partial u}{\partial t} = \widetilde{D}\left(\frac{\partial^2 u}{\partial r^2}\right) \qquad \text{... (5)}$$

resembling the equation for linear flow in one dimension, as (2). If the sphere is initially at a uniform concentration C_1 and the surface concentration is maintained constant at C_0, the boundary conditions can be expressed as

$$u = 0,\;\; r = 0,\;\; t > 0;$$

$$u = RC_0,\;\; r = R,\;\; t > 0;$$

$$u = rf(r),\;\; 0 < r < R,\;\; t = 0.$$

Then the solution (3) becomes

$$\frac{C - C_1}{C_0 - C_1} = 1 + \frac{2R}{\pi r}\sum_{n=1}^{\infty}\frac{(-1)^n}{n}\sin\left(\frac{n\pi r}{R}\right)\exp(-\widetilde{D}n^2\pi^2 t/R^2) \qquad \text{... (6)}$$

The total amount of diffusing substance entering or leaving the sphere, is given as the conversion fraction,

$$\frac{M_t}{M_\infty} = 1 - \frac{6}{\pi^2}\sum_{n=1}^{\infty}\frac{1}{n^2}\exp(-\widetilde{D}n^2\pi^2 t/R^2) \qquad \text{... (7)}$$

REFERENCES

1. Langof, L.; Ehrenfreund, E.; Lifshitz, E.; Micic, O. I.; Nozik, A. J., Continuous-Wave and Time-Resolved Optically Detected Magnetic Resonance Studies of Nonetched/Etched InP Nanocrystals. *J. Phys. Chem. B* **2002,** *106* (7), 1606-1612.
2. Mićić, O. I.; Nozik, A. J.; Lifshitz, E.; Rajh, T.; Poluektov, O. G.; Thurnauer, M. C., Electron and Hole Adducts Formed in Illuminated InP Colloidal Quantum Dots Studied by Electron Paramagnetic Resonance. *J. Phys. Chem. B* **2002,** *106* (17), 4390-4395.
3. Tessier, M. D.; De Nolf, K.; Dupont, D.; Sinnaeve, D.; De Roo, J.; Hens, Z., Aminophosphines: A Double Role in the Synthesis of Colloidal Indium Phosphide Quantum Dots. *J. Am. Chem. Soc.* **2016,** *138* (18), 5923-9.
4. Tessier, M. D.; Dupont, D.; De Nolf, K.; De Roo, J.; Hens, Z., Economic and Size-Tunable Synthesis of InP/ZnE (E = S, Se) Colloidal Quantum Dots. *Chem. Mater.* **2015,** *27* (13), 4893-4898.
5. Ginterseder, M.; Franke, D.; Perkinson, C. F.; Wang, L.; Hansen, E. C.; Bawendi, M. G., Scalable Synthesis of InAs Quantum Dots Mediated through Indium Redox Chemistry. *J. Am. Chem. Soc.* **2020,** *142* (9), 4088-4092.
6. Dirin, D. N.; Dreyfuss, S.; Bodnarchuk, M. I.; Nedelcu, G.; Papagiorgis, P.; Itskos, G.; Kovalenko, M. V., Lead halide perovskites and other metal halide complexes as inorganic capping ligands for colloidal nanocrystals. *J. Am. Chem. Soc.* **2014,** *136* (18), 6550-3.
7. Hudson, M. H.; Gupta, A.; Srivastava, V.; Janke, E. M.; Talapin, D. V., Synthesis of $In_{1-x}Ga_xP$ Quantum Dots in Lewis Basic Molten Salts: The Effects of Surface Chemistry, Reaction Conditions, and Molten Salt Composition. *J. Phys. Chem. C* **2022,** *126* (3), 1564-1580.
8. Ilavsky, J.; Jemian, P. R., Irena: tool suite for modeling and analysis of small-angle scattering. *J. Appl. Crystallogr.* **2009,** *42* (2), 347-353.
9. Hirel, P., Atomsk: A tool for manipulating and converting atomic data files. *Comput. Phys. Commun.* **2015,** *197*, 212-219.
10. Momma, K.; Izumi, F., VESTA 3 for three-dimensional visualization of crystal, volumetric and morphology data. *J. Appl. Crystallogr.* **2011,** *44* (6), 1272-1276.
11. Trigg, E. B., DebyeByPy. *GitHub repository* **2015**, https://github.com/etrigg/DebyeByPy.
12. Cromer, D. T.; Mann, J. B., X-ray scattering factors computed from numerical Hartree–Fock wave functions. *Acta Crystallogr. A* **1968,** *24* (2), 321-324.
13. Bawendi, M. G.; Kortan, A. R.; Steigerwald, M. L.; Brus, L. E., X-ray structural characterization of larger CdSe semiconductor clusters. *J. Chem. Phys.* **1989,** *91* (11), 7282-7290.
14. Uekermann F., F. S., Aradi B., Nanocut. **2009**.
15. Aradi, B., Nanocut. **2010-2012**.
16. Perdew, J. P.; Burke, K.; Ernzerhof, M., Generalized Gradient Approximation Made Simple. *Phys. Rev. Lett.* **1996,** *77* (18), 3865-3868.
17. Hamann, D. R.; Schlüter, M.; Chiang, C., Norm-Conserving Pseudopotentials. *Phys. Rev. Lett.* **1979,** *43* (20), 1494-1497.
18. Tsen, S. C. Y.; Crozier, P. A.; Liu, J., Lattice measurement and alloy compositions in metal and bimetallic nanoparticles. *Ultramicroscopy* **2003,** *98* (1), 63-72.

19. Natan, A., Fast 2D Peak Finder. *GitHub repository* **2021**, https://github.com/adinatan/fastpeakfind/releases/.
20. Crank, J., The Mathematics of Diffusion. 2nd ed.; Oxford University Press: London, 1975; pp 89-103.
